# Supplementary material for: Comparison of oral anticoagulants for stroke prevention in atrial fibrillation using the UK clinical practice research Datalink Aurum: A reference trial (ARISTOTLE) emulation study
Source: PLoS Med. 2024 Aug 29;21(8):e1004377. doi: 10.1371/journal.pmed.1004377 (PMC11361421; doi:10.1371/journal.pmed.1004377)
Supplement: S1 STROBE Checklist — (DOCX) [file pmed.1004377.s001.docx]

STROBE Statement—Checklist of items that should be included in reports of ***cohort studies***

|  | Item No | Recommendation | Page No |
| --- | --- | --- | --- |
| **Title and abstract** | 1 | (*a*) Indicate the study’s design with a commonly used term in the title or the abstract | Title |
|  |  | (*b*) Provide in the abstract an informative and balanced summary of what was done and what was found | Abstract |
| Introduction | | | |
| Background/rationale | 2 | Explain the scientific background and rationale for the investigation being reported | Introduction |
| Objectives | 3 | State specific objectives, including any prespecified hypotheses | Introduction, paragraph 5; Methods of Analysis, Benchmarking results against ARISTOTLE |
| Methods | | | |
| Study design | 4 | Present key elements of study design early in the paper | Abstract, Methods and Findings, paragraph 1; Materials and methods, Study design |
| Setting | 5 | Describe the setting, locations, and relevant dates, including periods of recruitment, exposure, follow-up, and data collection | Materials and methods, Setting/data sources |
| Participants | 6 | (*a*) Give the eligibility criteria, and the sources and methods of selection of participants. Describe methods of follow-up | Materials and methods, Patient Selection, Step 1 and Step 2; Table A2 in S3; Fig 2 |
|  |  | (*b*) For matched studies, give matching criteria and number of exposed and unexposed | Materials and methods, Patient Selection, Step 3; Fig 2;  Results of Propensity score matching |
| Variables | 7 | Clearly define all outcomes, exposures, predictors, potential confounders, and effect modifiers. Give diagnostic criteria, if applicable | Materials and methods, Diagnostic and therapeutic codelists and Exposures and outcomes;  Table 1; |
| Data sources/ measurement | 8* | For each variable of interest, give sources of data and details of methods of assessment (measurement). Describe comparability of assessment methods if there is more than one group | Exposures and outcomes; Table A2 in S3. |
| Bias | 9 | Describe any efforts to address potential sources of bias | Patient selection, Step 3; Methods of Analysis, Confounding and bias |
| Study size | 10 | Explain how the study size was arrived at | Fig 2;  Protocol in S2. |
| Quantitative variables | 11 | Explain how quantitative variables were handled in the analyses. If applicable, describe which groupings were chosen and why | Introduction, paragraph 2 for TTR |
| Statistical methods | 12 | (*a*) Describe all statistical methods, including those used to control for confounding | Patient selection, Step 3;  Methods of Analysis, paragraph 1;  Methods of Analysis, Sensitivity analyses |
|  |  | (*b*) Describe any methods used to examine subgroups and interactions | Methods of analysis, Supplementary analyses |
|  |  | (*c*) Explain how missing data were addressed | Methods of analysis, Missing data;  Table 2 row 6 on prior INR |
|  |  | (*d*) If applicable, explain how loss to follow-up was addressed | Methods of Analysis, paragraph 1; Methods of Analysis, Sensitivity analyses, paragraph 1. |
|  |  | (*e*) Describe any sensitivity analyses | Methods, Sensitivity analyses. |
| Results | | |  |
| Participants | 13* | (a) Report numbers of individuals at each stage of study—eg numbers potentially eligible, examined for eligibility, confirmed eligible, included in the study, completing follow-up, and analysed | Fig 2 |
|  |  | (b) Give reasons for non-participation at each stage | Fig 2 |
|  |  | (c) Consider use of a flow diagram | Fig 2 |
| Descriptive data | 14* | (a) Give characteristics of study participants (eg demographic, clinical, social) and information on exposures and potential confounders | Table 3, Table A8 in S3 |
|  |  | (b) Indicate number of participants with missing data for each variable of interest | Table 3 |
|  |  | (c) Summarise follow-up time (eg, average and total amount) | Main results, paragraph 1; Table A3 in S3, Table A5 in S3 |
| Outcome data | 15* | Report numbers of outcome events or summary measures over time | Tables A3 and A5 in S3 |

| Main results | 16 | (*a*) Give unadjusted estimates and, if applicable, confounder-adjusted estimates and their precision (eg, 95% confidence interval). Make clear which confounders were adjusted for and why they were included | Main results |
| --- | --- | --- | --- |
|  |  | (*b*) Report category boundaries when continuous variables were categorized | Table 3 shows categorisation of variables; Methods of analyses, Supplementary analyses |
|  |  | (*c*) If relevant, consider translating estimates of relative risk into absolute risk for a meaningful time period | Not appropriate for non-inferior results. Absolute event rates (%/yr) provided in Fig3 and Fig4. |
| Other analyses | 17 | Report other analyses done—eg analyses of subgroups and interactions, and sensitivity analyses | Results, Analysis of impact of warfarin time in therapeutic range (TTR); Results, Analysis of apixaban dose-adjustment; Results, Sensitivity analyses. |
| Discussion | | | |
| Key results | 18 | Summarise key results with reference to study objectives | Discussion paragraph 1; |
| Limitations | 19 | Discuss limitations of the study, taking into account sources of potential bias or imprecision. Discuss both direction and magnitude of any potential bias | Sensitivity Analyses; Limitations |
| Interpretation | 20 | Give a cautious overall interpretation of results considering objectives, limitations, multiplicity of analyses, results from similar studies, and other relevant evidence | Discussion paragraphs 1 and 2 |
| Generalisability | 21 | Discuss the generalisability (external validity) of the study results | Limitations, paragraph 3; Conclusions paragraphs 1 and 2 |
| Other information | | | |
| Funding | 22 | Give the source of funding and the role of the funders for the present study and, if applicable, for the original study on which the present article is based | Funding statement |

*Give information separately for exposed and unexposed groups.

**Note:** An Explanation and Elaboration article discusses each checklist item and gives methodological background and published examples of transparent reporting. The STROBE checklist is best used in conjunction with this article (freely available on the Web sites of PLoS Medicine at http://www.plosmedicine.org/, Annals of Internal Medicine at http://www.annals.org/, and Epidemiology at http://www.epidem.com/). Information on the STROBE Initiative is available at http://www.strobe-statement.org.
