# Supplementary material for: Comparison of oral anticoagulants for stroke prevention in atrial fibrillation using the UK clinical practice research Datalink Aurum: A reference trial (ARISTOTLE) emulation study
Source: PLoS Med. 2024 Aug 29;21(8):e1004377. doi: 10.1371/journal.pmed.1004377 (PMC11361421; doi:10.1371/journal.pmed.1004377)
Supplement: S1 ISAC Protocol — (PDF) [file pmed.1004377.s002.pdf]

ISAC EVALUATION OF PROTOCOLS FOR RESEARCH INVOLVING CPRD DATA

FEEDBACK TO APPLICANTS

**CONFIDENTIAL**	*by e-mail*
**PROTOCOL NO:**	19_066R
**PROTOCOL TITLE:**	Use of non-interventional data for determining the real-world effectiveness of anticoagulation medication for stroke prevention in a clinical trial analogous population
**APPLICANT:**	Dr Kevin Wing<br>London School of Hygiene & Tropical Medicine<br>[redacted]

**APPROVED**	**APPROVED WITH COMMENTS (resubmission not required)**	**REVISION/ RESUBMISSION REQUESTED**	**REJECTED**
☐	☐	☐	☐

INSTRUCTIONS:

Protocols with an outcome of 'Approved' or 'Approved with comments' do not require resubmission to the ISAC.

REVIEWER COMMENTS:

APPLICANT FEEDBACK:

**DATE OF ISAC FEEDBACK:**	19/09/19
**DATE OF APPLICANT FEEDBACK:**	

For protocols approved from 01 April 2014 onwards, applicants are required to include the ISAC protocol in their journal submission with a statement in the manuscript indicating that it had been approved by the ISAC (with the reference number) and made available to the journal reviewers. If the protocol was subject to any amendments, the last amended version should be the one submitted.

Guidance on resubmitting applications, or making amendments to approved protocols, can be found on the CPRD website at https://cprd.com/research-applications.

Medicines & Healthcare products Regulatory Agency

CPRD

INDEPENDENT SCIENTIFIC ADVISORY COMMITTEE (ISAC) PROTOCOL APPLICATION FORM

PART 1: APPLICATION FORM

IMPORTANT

Both parts of this application must be completed in accordance with the guidance note 'Completion of the ISAC Protocol Application Form', which can be found on the CPRD website cprd.com/research-applications

**FOR ISAC USE ONLY**	
**Protocol No. -**	**Submission date -**

GENERAL INFORMATION ABOUT THE PROPOSED RESEARCH STUDY

1. Study Title (Max. 255 characters)

Use of non-interventional data for determining the real-world effectiveness of anticoagulation medication for stroke prevention in a clinical trial analogous population

2. Research Area (place '**X**' in all boxes that apply)

Drug Safety	X	Economics	
Drug Utilisation		Pharmacoeconomics	
Drug Effectiveness	X	Pharmacoepidemiology	X
Disease Epidemiology		Methodological	X
Health Services Delivery			

3. Chief Investigator

Title:	Dr
Full name:	Kevin Wing
Job title:	Assistant Professor of Epidemiology
Affiliation/organisation:	London School of Hygiene & Tropical Medicine
Email address:	[redacted]
CV Number (if applicable):	

4. Corresponding Applicant

Title:	Ms
Full name:	Emma Powell
Job title:	Research Degree Student
Affiliation/organisation:	London School of Hygiene & Tropical Medicine
Email address:	[redacted]
CV Number (if applicable):	

5. List of all investigators/collaborators

Title:	Ms
Full name:	Emma Powell
Job title:	Research Degree Student
Affiliation/organisation:	London School of Hygiene & Tropical Medicine
Email address:	[redacted]
CV Number (if applicable):	
Will this person be analysing the data? (Y/N)	Y

Title:	Dr
Full name:	Kevin Wing
Job title:	Assistant Professor of Epidemiology
Affiliation/organisation:	London School of Hygiene & Tropical Medicine
Email address:	[redacted]
CV Number (if applicable):	
Will this person be analysing the data? (Y/N)	N

Title:	Dr
Full name:	Ian Douglas
Job title:	Associate Professor of Pharmacoepidemiology
Affiliation/organisation:	London School of Hygiene & Tropical Medicine
Email address:	[redacted]
CV Number (if applicable):	
Will this person be analysing the data? (Y/N)	N

Title:	Ms
Full name:	Usha Gungabissoon
Job title:	Director, Epidemiology
Affiliation/organisation:	GlaxoSmithKline
Email address:	[redacted]
CV Number (if applicable):	
Will this person be analysing the data? (Y/N)	N

Title:	Prof
Full name:	Liam Smeeth
Job title:	Professor of Clinical Epidemiology
Affiliation/organisation:	London School of Hygiene & Tropical Medicine
Email address:	[redacted]
CV Number (if applicable):	
Will this person be analysing the data? (Y/N)	N

[Add more investigators/collaborators as necessary by copy and pasting a new table for each investigator/collaborator]

6. Experience/expertise available

List below the member(s) of the research team who have experience with CPRD data.

**Name:**	**Protocol Number/s:**
Kevin Wing, Ian Douglas, Usha Gungabissoon, Liam Smeeth	>50 protocols

List below the member(s) of the research team who have statistical expertise.

Name(s):
Emma Powell

List below the member(s) of the research team who have experience of handling large datasets (greater than 1 million records).

Name(s):
Kevin Wing
Ian Douglas

List below the member(s) of the research team, or supporting the research team, who have experience of practicing in UK primary care.

Name(s):
Liam Smeeth

ACCESS TO THE DATA

7. Sponsor of the study

Institution/Organisation:	Medical Research Council
Address:	Polaris House, North Star Avenue, Swindon, SN2 1FL, United Kingdom

8. Funding source for the study

Same as Sponsor?	Yes	X	No	
Institution/Organisation:	Medical Research Council			
Address:	Polaris House, North Star Avenue, Swindon, SN2 1FL, United Kingdom			

9. Institution conducting the research

Same as Sponsor?	Yes		No	X
Institution/Organisation:	London School of Hygiene & Tropical Medicine			
Address:	Keppel Street, London, WC1E 7HT, United Kingdom			

10. Data Access Arrangements

Indicate with an '**X**' the method that will be used to access the data for this study:

Study-specific Dataset Agreement	

Institutional Multi-study Licence	X
Institution Name	London School of Hygiene & Tropical Medicine
Institution Address	Keppel Street, London, WC1E 7HT, United Kingdom

Will the dataset be extracted by CPRD?

Yes		No	X

If yes, provide the reference number:

11. Data Processor(s):

Processing	X

Accessing	X	
Storing	X	
Processing area (UK/EEA/Worldwide)		UK
Organisation name		London School of Hygiene & Tropical Medicine
Organisation address		Keppel Street, London, WC1E 7HT, United Kingdom

Processing		
Accessing		
Storing		
Processing area (UK/EEA/Worldwide)		
Organisation name		
Organisation address		

[Add more processors as necessary by copy and pasting a new table for each processor]

INFORMATION ON DATA

12. Primary care data (place '**X**' in all boxes that apply)

CPRD GOLD	X	CPRD Aurum	X

13. Please select any linked data or data products being requested

Patient Level Data (place '**X**' in all boxes that apply)

ONS Death Registration Data	X	CPRD Mother Baby Link	
HES Admitted Patient Care	X	Pregnancy Register	
HES Outpatient		NCRAS (National Cancer Registration and Analysis Service) Cancer Registration Data	
HES Accident and Emergency		NCRAS Cancer Patient Experience Survey (CPES) data	
HES Diagnostic Imaging Dataset		NCRAS Systemic Anti-Cancer Treatment (SACT) data	
HES PROMS (Patient Reported Outcomes Measure)		NCRAS National Radiotherapy Dataset (RTDS) data	
		Mental Health Services Data Set (MHDS)	

Area Level Data (place '**X**' in all boxes that apply)

**Practice level (UK)**		**Patient level (England only)**	
Practice Level Index of Multiple Deprivation (Standard)	X	Patient Level Index of Multiple Deprivation	X
Practice Level Index of Multiple Deprivation (Non-standard)		Patient Level Townsend Score	
Practice Level Index of Multiple Deprivation Domains (Non-standard)			
Practice Level Carstairs Index for 2011 Census (Excluding Northern Ireland) (Standard)			
2011 Rural-Urban Classification at LSOA level (Non-standard)			

Reference number (where applicable):
**14. Are you requesting linkage to a dataset not listed above?**<br><br>Yes [ ] No [X]<br><br>If yes, provide the reference number:
**15. Does any person named in this application already have access to any of these data in a patient identifiable form, or associated with an identifiable patient index?**<br><br>Yes [ ] No [X]<br><br>If yes, provide further details:
**VALIDATION/VERIFICATION**
**16. Does this protocol describe an observational study using purely CPRD data?**<br><br>Yes [X] No [ ]
**17. Does this protocol involve requesting any additional information from GPs, or contact with patients?**<br><br>Yes [ ] No [X]<br><br>If yes, provide the reference number:

PART 2: PROTOCOL INFORMATION

**Applicants must complete all sections listed below**<br>**Sections which do not apply should be completed as '*Not Applicable*' and justification provided**
**A. Study Title (Max. 255 characters)**<br>Use of non-interventional data for determining the effectiveness of anticoagulation medication for stroke prevention
**B. Lay Summary (Max. 250 words)**<br>Atrial Fibrillation (AF) is a heart condition in which patients have an irregular heartbeat. Patients with AF are at a higher risk of stroke and may be prescribed a type of medication called anticoagulants to reduce the risk of stroke.<br><br>Treatment guidelines for anticoagulants are based on the results of randomised clinical trials which have very strict entry criteria. This means that many people with AF who are prescribed these drugs by their GP could be quite different to patients studied in clinical trials. The types of patients who are usually not allowed to take part in clinical trials include people with existing medical conditions, people with no other stroke risk factors, and pregnant patients. Patients included in anticoagulant clinical trials may therefore not be representative of the patients who are prescribed these drugs in clinical practice.<br><br>In this study we will look at how well a specific anticoagulant medicine called apixaban works compared with another treatment called warfarin in people who would have been excluded from a landmark anticoagulant trial (the ARISTOTLE trial), in order to help improve treatment guidelines for these people. We will use information routinely collected by GPs and hospitals to assess the impact of apixaban and warfarin in preventing stroke and blood vessel blockage. First we will see how well these treatments work when prescribed to people that are similar to those included in the ARISTOTLE trial. Then we will see how well the treatments work when prescribed to patient groups excluded from this trial.
**C. Technical Summary (Max. 300 words)**<br>Patients with atrial fibrillation (AF) are at a greatly increased risk of stroke; prophylactic treatment with anticoagulation medication reduces this risk. In the last decade several direct oral anticoagulants (DOACs) have been approved providing an alternative to the standard treatment warfarin which has many drug and dietary interactions and requires onerous monitoring. Treatment guidelines for AF patients are based on the results from randomised controlled trials (RCT).<br><br>There is increasing interest in the effectiveness of medications in routine clinical practice to confirm trial results and estimate drug effectiveness in patient groups excluded from or underrepresented in clinical trials. There is however some uncertainty about the suitability of using non-interventional data to address questions about drug effectiveness and on the most suitable methods to be used. The aims of this study are to attempt to measure the association between anticoagulation treatments for stroke prevention in AF using electronic health records (EHRs) and to develop a methodological framework for using observational EHRs to answer questions about DOACs among patients excluded from or underrepresented in the RCTs.<br><br>This study will use individual patient data from ARISTOTLE[1], a pivotal trial conducted 2006-2011 in 18,201 patients that demonstrated superiority of the DOAC apixaban compared with warfarin in prevention of stroke. The individual patient data will be used to match to UK NHS patients with anonymised routinely-collected EHRs from CPRD. Analysis of drug effectiveness in this cohort will help determine whether EHR data are suitable for this kind of research question. Selecting EHR patients similar to trial patients will remove much of the variability in baseline risk of the study outcomes between trial participants and EHR patients. If we can demonstrate replication of trial results in our ARISTOTLE-analogous cohort, the same methodology will be used to determine drug effectiveness in patient groups underrepresented in the trial.
**D. Outcomes to be Measured**<br>Time to event: stroke, systemic embolism, myocardial infarction, all-cause death, major bleeding

E. Objectives, Specific Aims and Rationale

Aim 1: To measure the association between anticoagulation treatments for stroke prevention in AF and time to stroke, systemic embolism, myocardial infarction, major bleeding, and mortality amongst an ARISTOTLE-analogous cohort of patients from UK electronic health records (EHR).

Aim 2: To develop a methodological framework with in-built validation, for using observational electronic health records to answer questions about DOAC risks and benefits in patients excluded from or underrepresented in the RCTs.

Objectives

Objective 1. Check comparability of EHR data and robustness of methods for measuring AF stroke prevention medication effectiveness in EHR data by comparing with ARISTOTLE results.

We will obtain fully anonymised individual patient data from the pivotal ARISTOTLE trial. A group of individuals with similar characteristics will be selected from EHR data based on medical history, prescription data, and baseline characteristics. The ARISTOTLE trial measured the efficacy of apixaban vs warfarin for the prevention of stroke, systemic embolism, myocardial infarction, and mortality and safety (major bleeding) amongst people with AF and at least one risk factor for stroke. Individual trial participants will be matched with similar people in the anonymised EHR databases the Clinical Practice Research Datalink (CPRD) Gold and CPRD Aurum in order to create an ARISTOTLE-analogous cohort within EHRs. The individual trial participant information is being obtained from Bristol Myers-Squibb. Using cohort methodology, estimates of the effect of apixaban vs warfarin on stroke, systemic embolism, myocardial infarction, major bleeding and mortality in the CPRD cohort of ARISTOTLE analogous patients will then be measured. The results will be compared with the ARISTOTLE findings to determine the utility of CPRD records for measuring medication effectiveness in AF. This objective will provide a methodological framework for measuring drug effectiveness in people with AF, using observational data from CPRD.

Objective 2. Extension of trial findings: Measure AF treatment effects in patients excluded from ARISTOTLE

Using the methodological template developed in Objective 1, we will determine the effect of apixaban vs warfarin in prevention of stroke, systemic embolism, myocardial infarction, major bleeding and mortality in people with AF not eligible for the ARISTOTLE study (most importantly people with substantial comorbidity), and look separately at important subgroups e.g. those with and without underlying cardiovascular disease.

Objective 3. Comparative effectiveness: Compare treatment effectiveness between multiple individual anticoagulants in all anticoagulant recipients (no eligibility criteria other than diagnosis of AF)

Using the methodological template developed in Objective 1, we will compare time to stroke, systemic embolism, myocardial infarction, major bleeding, and death based on prescribed treatments:
a) warfarin
b) apixaban
c) rivaroxaban
d) dabigatran
Apixaban will be compared with warfarin then all other DOACs compared with apixaban.

Rationale

AF treatment guidelines are largely informed by randomised controlled trial (RCT) results, but we do not know if these findings apply to large patient populations not studied in trials. Apixaban is one of the most widely used anticoagulants used in stroke prevention in AF. It was studied in a large randomised trial (ARISTOTLE), but we don't know the effects of treatment in important patient groups who were not studied. Some were excluded from AF stroke prevention trials in general (e.g. those with mechanical heart valves and those with substantial comorbidity) and some are under-represented (e.g. elderly patients), meaning conclusions about these groups are difficult to make. The results we generate will firstly tell us if EHR data are suitable for this purpose. If so, our findings will aid patients, prescribers and policy makers in better understanding the benefits and risks of different anticoagulants for stroke prophylaxis in AF rather than assuming that the trial estimates are applicable to all patients.

F. Study Background

Atrial fibrillation (AF) is a heart condition in which the patient experiences a rapid and irregular heartbeat caused by electrical signals firing from multiple locations in the atria. Although patients may be asymptomatic , symptoms such as heart palpitations, fainting, light-headedness, and shortness of breath are reported. The prevalence of AF is estimated to be around 3%[2] and increases with age from 0.2% in people aged 45-54 years to 8.0% in those 75 and older[3]. The lack of organised atrial contraction in AF can lead to blood stagnating in the left atrium or left atrial appendage and the formation of thrombi. Should a thrombus move from the heart through the body this can cause systemic embolism or stroke; consequently patients with AF have a five fold higher risk of stroke. It is recommended that AF patients receive prophylactic treatment with anticoagulation medication to reduce the risk of stroke. The previous standard anticoagulation treatment for this indication, warfarin, has many treatment interactions and requires frequent monitoring and dose adjustments to stay within the therapeutic range of anticoagulant action as summarised by Hirsh et al[4]: Warfarin has interactions with a wide range of drugs such as metronidazole which inhibits warfarin clearance, barbiturates and carbamazepine which increase hepatic warfarin, and aspirin which increases the risk of bleeding. Diet also interacts with response to warfarin with increased intake of vitamin K (present in green vegetables) leading to a reduction in the anticoagulant response to warfarin. Genetics influences the warfarin dose-response relationship most notably in common mutations in coding for cytochrome P450 (the family of enzymes responsible for warfarin metabolism). Warfarin therapy is monitored by calculating a patient’s International Normalised Ratio (INR), a standardised measurement of the time taken for blood to clot. Typically for AF the patient must maintain INR at a therapeutic range between 2.0 and 3.0 with INR values below 2 putting a patient at higher risk of stroke and levels above 3 resulting in a higher risk of bleeding. On initiation of therapy INR is checked daily until in therapeutic range, then 3 times weekly for 2 weeks, then less often, according to the stability of the results. Given the challenge in maintaining INR in therapeutic range and the complex safety profile of warfarin it was hoped that the introduction of the direct acting oral anticoagulants (DOACs) would provide a safer and easier to manage long term anticoagulation therapy for AF patients. The pivotal trial of the DOAC apixaban for this indication, ARISTOTLE, demonstrated superiority over warfarin for both the primary efficacy (prevention of stroke) and safety (major bleeding) outcomes.

Apixaban was licensed based on the results of the pivotal trial ARISTOTLE, a randomised controlled trial (RCT). ARISTOTLE had eligibility criteria that patients had to meet to be included in the trial, thus limiting the generalisability of the results of the trial. As a result, evidence on treatment effect is lacking for patients who would not have met the ARISTOTLE eligibility criteria such as individuals with a mechanical heart valve, those at increased bleeding risk, and individuals with severe comorbid conditions. The regulatory environment now demands evidence of treatment effectiveness outside the confines of randomised trials. Non-interventional data sources have the potential to overcome many of the RCT limitations given that they contain data for a wide spectrum of patients treated with the drug in routine care including patients who would have been excluded from trials. Data collected as a standard part of patient care such as electronic healthcare record (EHRs) provide a valuable opportunity to obtain evidence on the effectiveness of apixaban in a routine care setting. A key problem with non-interventional studies using these data is that the absence of randomisation leaves them highly susceptible to confounding (with confounding by indication a particular problem), making it difficult to have confidence in the results. By contrast matching to individual patient data from ARISTOTLE and then using novel methods for matching within EHR treatment groups should result in an EHR population similar to the trial population that is well balanced by treatment group. If successful, the estimates of effectiveness and safety of apixaban obtained from this approach should then be comparable with the ARISTOTLE results. If non-interventional data can be successfully used to approximate the findings of ARISTOTLE then they may be reliable to estimate effects in under studied AF patient groups. This project will involve testing whether EHR data can find results compatible with the ARISTOTLE trial results while developing optimal methodology for studying anticoagulants in stroke prophylaxis. This methodology can then be applied to under studied AF patient groups.

G. Study Type

Hypothesis testing

H. Study Design

This is a historical cohort study.

The cohort study design allows measurement of the effects of prescribing apixaban vs warfarin for prevention of stroke and systemic embolism in AF on key efficacy and safety outcomes..

**I. Feasibility counts**
ARISTOTLE inclusion and exclusion criteria were extracted from the trial protocol. Read code and medication codelists were created for the inclusion and exclusion criteria. The criteria were applied to a January 2018 extract of CPRD Gold patients prescribed apixaban. Overall out of 13 332 patients with a prescription for apixaban and diagnosis of AF 63% (8 407) were trial-eligible. Trial criteria were also applied to patients with a prescription for warfarin in the period 01 January 2013 to 31 January 2018 and a diagnosis of AF (68 113 patients). Of these patients 45 435 (62.3%) were eligible according to trial criteria.
**J. Sample size considerations**
We will include all eligible patients registered in the CPRD and who meet the trial criteria. In ARISTOTLE there were 9120 subjects in the apixaban arm therefore it was estimated a minimum of 15,000 EHR patients exposed to apixaban were needed for matching to be feasible. It was unlikely there would be enough patients in CPRD Gold alone for the project given that only ~8400 patients were eligible in the January 2018 extract. The CPRD Aurum database (June 2019 extract) contained 29,578 patients with both an atrial fibrillation diagnosis and a prescription for apixaban; of these patients 23,526 were not registered in practices that had previously contributed data to CPRD Gold. Using the assumption that the proportion of Aurum patients who would meet the ARISTOTLE trial eligibility criteria would be similar to the proportion of Gold patients (~60%) gave an estimate of 14,115 trial eligible Aurum patients in the apixaban arm. Combining the Gold and Aurum cohorts is therefore estimated to give >22,000 unique trial-eligible EHR apixaban patients.
**K. Planned use of linked data (if applicable):**
We intend to use CPRD data linked with HES in patient data to enable optimal stroke, MI, and major bleeding ascertainment, and ONS mortality data to determine deaths. When matching CPRD patients we plan to include the practice level deprivation level index as a matching variable because socioeconomic status is predicted to influence the likelihood of the primary study outcome of stroke.

L. Definition of the Study population

For all objectives two datasets of UK primary care data will be combined: CPRD Gold and CPRD Aurum. Patients with a prescription for an oral anticoagulant in the time period 01 January 2013 to 31 January 2019 (exact cut-off date dependent on date of final data extracts) and a prior diagnosis of atrial fibrillation will be selected as the EHR cohort. Apixaban gained UK marketing authorisation in January 2013 for the indication of prevention of stroke and systemic embolism in AF patients; the minimum date is set to capture all patients prescribed apixaban for this indication in the UK. A previous study validated the use of diagnostic read codes for identifying patients with AF in CPRD by sending surveys to GPs and found a confirmation rate of 98% among patients originally identified with AF codes[5].

Objective 1

Step 1: We will select all (HES and ONS linked) patients in the EHR cohort who would have met the following eligibility criteria for inclusion in the ARISTOTLE study, at least 6 months after patient registration in an up to standard practice: or 6 months post-UTS date, whichever is later:

- a diagnosis of AF,
- age over 18 years,
- at least one of the following risk factors for stroke: diagnosis of congestive heart failure, hypertension requiring pharmacological treatment, age greater than 75, diabetes mellitus, and prior stroke or systemic embolism,
- no AF due to reversible causes (e.g. thyrotoxicosis, pericarditis),
- no clinically significant (moderate or severe) mitral stenosis, in EHRs clinical significance and severity are not consistently recorded therefore a diagnosis of mitral stenosis will be sufficient to exclude a patient,
- no increased bleeding risk that is believed to be a contraindication to oral anticoagulation (e.g. previous intracranial haemorrhage),
- no conditions other than AF that require chronic anticoagulation (e.g. prosthetic mechanical heart valve),
- no persistent, uncontrolled hypertension (systolic BP > 180 mm Hg, or diastolic BP > 100 mm Hg), in EHRs this will be implemented by excluding patients whose latest blood pressure reading in the 6 months prior to the index date is over the systolic or diastolic blood pressure limit
- no active infective endocarditis,
- no concomitant treatment with aspirin > 165 mg/day,
- no simultaneous treatment with both aspirin and a thienopyridine (e.g., clopidogrel, ticlopidine),
- no severe comorbid condition with life expectancy of ≤ 1 year,
- no active alcohol or drug abuse, or significant psychosocial difficulties (e.g., psychosis, dementia),
- no recent ischemic stroke (within 7 days),
- no severe renal insufficiency (serum creatinine > 2.5 mg/dL or a calculated creatinine clearance < 25 mL/min),
- no ALT or AST > 2X ULN or a Total Bilirubin ≥ 1.5X ULN (unless an alternative causative factor such as Gilbert's syndrome is identified),
- no platelet count ≤ 100,000/ mm^{3},
- no haemoglobin < 9 g/dL,
- no prior exposure to apixaban,
- no women who are pregnant or breastfeeding

For the 3 criteria involving patient laboratory results (renal insufficiency, low platelet count, and low haemoglobin) a patient will be excluded if their last test result in the 90 days prior to the index date meets the exclusion criteria.

Step 2: Next we will determine if/when these patients received apixaban or warfarin. Individuals in EHR who have more than one warfarin eligibility period within their record will be able to contribute more than once to the pool of warfarin subjects (with the covariates and person-time contributed unique to the specific eligibility period) as long as they have no past apixaban exposure.

Step 3: Having obtained individual level patient data for ARISTOTLE participants from Bristol Myers-Squibb we will then match each ARISTOTLE apixaban participant 1:1 with the closest available apixaban patient record in our EHR pool. We will consider matching on a selection of the following ARISTOTLE baseline characteristics:

- age
- sex
- body mass index
- systolic blood pressure (mmHg)
- history of congestive heart failure or left ventricular systolic dysfunction

- hypertension requiring pharmacological treatment
- diabetes mellitus
- prior stroke or TIA or thromboembolism
- smoking status [current smoker, former smoker, or never smoked]
- alcohol consumption [none, low, moderate, heavy]
- renal impairment [severe, moderate, mild, normal based on the CrCL value]
- prior VKA/warfarin use
- concomitant treatment with:
  anticoagulant/VKA use other than warfarin or apixaban
  aspirin
  antiplatelets
  NSAIDs
  lipid lowering drug therapy
  CYP3A4 inhibitors

We anticipate matching all or the majority of ARISTOTLE apixaban subjects with an EHR patient, giving us a pool of ARISTOTLE-analogous apixaban patients, with similar baseline characteristics as ARISTOTLE subjects at the point of randomisation (n~9,000).

The variables selected as potential matching variables are those known or suspected to influence the likelihood of the outcomes of interest. The exact selection of matching variables will depend on the quality and completeness of the data available and a balance will be struck between the matched sample size and sample balance. Variables may be grouped together to increase the sample size, for example by grouping concomitant medications that increase bleeding risk. Continuous variables may be coarsened by splitting into appropriate categories. A procedure will be employed to facilitate selection of a matched cohort, for example via coarsened exact matching[6] for example by use of the %CEM SAS macro[7]. Coarsened Exact Matching is a nonparametric matching method that has been found to give estimates of casual effects with lower variance and bias for a given sample size compared with other commonly used methods of matching[8].

Figure 1: Assembly of Matched Trial-analogous Cohort of EHR Patients

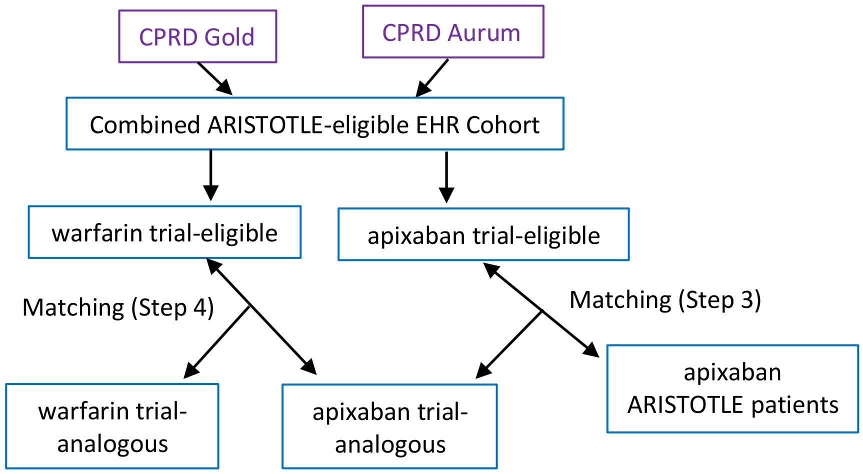

Step 4: The resulting trial matched sample of EHR apixaban exposed subjects will be matched to the warfarin ARISTOTLE-eligible EHR subjects (Figure 1) using a matching method such as propensity score matching (PSM), or CEM (with the final method selected based upon method giving the optimal sample size versus balance). Where an individual from EHR has multiple warfarin “eligibility periods” that can be matched to an apixaban trial matched subject, the EHR characteristics that will be matched on will be those from the beginning of the specific eligibility period. The covariates for consideration in the matching between EHR treatment arms or construction of a PS model will include the variables listed above used in in step 3 along with additional EHR variables such as data source (CPRD Gold or Aurum), socioeconomic status, and comorbidities. The hazard ratio for the outcomes of interest (listed in section N) will then be calculated. Each apixaban patient from the ARISTOTLE-eligible EHR

patients will be matched 1:1 with the warfarin EHR patient with the closest match giving a trial-analogous analysis cohort of ~18,000.

A patient may be exposed to warfarin followed by apixaban in the time period of interest and the patient be trial eligible in both treatment periods; in such a situation both patient treatment periods may be included in the EHR treatment groups with the restriction that a patient must not be matched to themselves. Given the trial exclusion criteria that a patient may not have been previously exposed to apixaban, any warfarin treatment periods after apixaban exposure would not be eligible for inclusion in the warfarin EHR cohort.

Objective 2: we will select patients who would not have been included in the ARISTOTLE trial (and therefore would not have been included in the Objective 1 cohort) based on their age, stroke risk factors, or presence of substantial comorbidity. Specifically, this will be patients with an AF diagnosis in the EHR cohort meeting these additional criteria:

- age >77 years (although elderly patients were not excluded the maximum age at first dose in ARISTOTLE was 76),

OR

- no evidence of at least one additional risk factor for stroke

OR

- AF due to reversible causes

OR

- evidence of drug/alcohol abuse

OR

- severe comorbid condition: ARISTOTLE required patients to be excluded from the trial if they had serious disease with a likelihood of causing death within 1 year or reasons making participation unpractical (such as dementia).

In these special patient populations the same outcomes as objective 1 will be assessed as described in section N.

Objective 3: we will select all patients with AF with a prescription for any anticoagulant in the treatment period from the set of treatments: apixaban, warfarin, rivaroxaban, dabigatran. For this objective all outcomes listed in section N will be assessed. Patients will be stratified on whether they would have met the ARISTOTLE trial criteria.

M. Selection of comparison group(s) or controls

For objectives 1 and 2 the comparison group consists of the patients prescribed warfarin with a diagnosis of AF meeting the eligibility conditions described in section L. For objective 3 apixaban is compared to each other treatment group (warfarin, rivaroxaban, dabigatran).

N. Exposures, Outcomes and Covariates

The exposures of interest are apixaban, warfarin, rivaroxaban, and dabigatran (any dose for each exposure).
The individual effectiveness outcomes:

- stroke
- systemic embolism
- myocardial infarction
- all cause death

The safety outcome for the study:

- major bleeding (bleeding requiring transfusion, bleeding at a critical site, bleeding requiring attendance at hospital, or fatal bleeding)

For all outcomes we make the assumption that if there is no record of an outcome then the outcome did not occur.

The EHR ARISTOTLE-eligible patients prescribed apixaban will be matched to the ARISTOTLE apixaban patients on the variables listed in section L (step 3 of objective 1), with value taken at baseline (value at time of first dose or latest measurement/data recorded prior to first dose).

When matching between the treatment arms within the EHR cohort of patients the variables to be considered for inclusion in the matching algorithm include those listed in section L used in matching from the EHR treatment arm to the trial patient data in addition to EHR variables such as data source (CPRD Gold or Aurum), socioeconomic status, and comorbidities.

O. Data/ Statistical Analysis

Data mapping

For all 3 objectives the data source will consist of two datasets of UK real world clinical data which will be combined: CPRD Gold and CPRD Aurum. The two datasets will be mapped to a common data model based on adapted Clinical Data Interchange Standards Consortium (CDSIC) standards[9]. Duplicate entries of patients – where a patient is present in more than one dataset – will be removed with the following rule: keep in CPRD Aurum above CPRD Gold. CPRD Aurum provides a dataset listing practices in CPRD Aurum which have previously contributed data to CPRD Gold; this dataset will be used to exclude data from practices with eligible patients in CPRD Gold where the patient data is also recorded in Aurum. Only data of interest to the study will be mapped and used: patient demographics, diagnoses, clinical events, and recorded symptoms, therapies prescribed, lab results, and vital signs.

Primary Analysis

o Population

The ARISTOTLE trial used an intent to treat (ITT) approach for the primary efficacy analysis, and an on-treatment approach for the sensitivity analyses and safety outcomes. To perform an equivalent analysis with the EHR data the following analysis populations will be used for all objectives:
<u>Prescribed Population</u>: all patients who were prescribed a treatment, regardless of future changes to treatment. When summarizing data using this population, subjects are categorized according to the As Prescribed group.
<u>On-treatment Population</u>: all patients who were prescribed a treatment. In the case of patients discontinuing or switching treatment, data will be included up to and including their derived date of last dose of the initially prescribed treatment.

o Censoring

<u>Index date</u>

The index date for the EHR cohort will be the date of the patient's first prescription of apixaban or warfarin (for objectives 1 and 2), or for objective 3 first prescription of apixaban, warfarin, rivaroxaban, or dabigatran on or after the date the patient first met the eligibility criteria for the trial in the treatment period of interest

<u>Date of last dose (all objectives)</u>

The date of last dose will be estimated using the subject's date of prescription, number of tablets prescribed, and daily dose. Where there are missing values for the number of tablets prescribed or daily dose suitable values will be used to replace these, for example by substituting with the median or modal value. To allow for stockpiling of tablets and less than 100% adherence we will add 30 days after the apparent end of treatment when deriving date of last dose.

<u>Primary censoring scheme:</u>

Patients will be censored at the earliest of: outcome of interest, death date, 'transferred out date', 'last collection date', or 5 years after the index date. The 5-year limit reflects the maximum possible follow-up for a subject in the ARISTOTLE trial. Conclusions regarding noninferiority or superiority will be based on the results of the analyses using this censoring scheme as this most closely resembles the ARISTOTLE analysis plan.

<u>Supportive censoring scheme:</u>

Patients will be censored at the earliest of: outcome of interest, death date, 'transferred out date', 'last collection date', 5 years after the index date, or the derived last date of study drug. By censoring around the time of last study drug this scheme should include only events likely to be due to the drug taken.

o Primary outcome

The primary efficacy endpoint will be the time to first occurrence of confirmed stroke (ischemic, hemorrhagic, or of unspecified type), or systemic embolism during the study, regardless of whether the subject is receiving treatment at the time of the event (i.e. using the primary censoring scheme).
Comparisons will be made according to prescribed treatment (apixaban vs warfarin) for time to stroke/SE.
This analysis approach will be used as the primary analysis for all 3 objectives.

o Descriptive analyses

<u>Demographic and Baseline Characteristics</u>

Frequency distribution and summary statistics for demographic and baseline variables will be presented by treatment group for the EHR patients, both before and after matching steps.

<u>Treatment Switching and Discontinuation</u>

As the primary analysis accounts neither for treatment switching nor for treatment discontinuation and does not capture that these may be unfavourable outcomes both the proportion of patients discontinuing treatment and time to treatment discontinuation will be tabulated by prescribed treatment.

o Regression model
Hazard ratio (apixaban/warfarin) comparing the event rate (%/yr).
Interpretation: This estimand targets the treatment-policy effect of treatment initiation of apixaban vs initiation or continuation of warfarin on the time to first stroke or systemic embolism.
All time to event endpoints will be analysed using a Cox proportional hazards model including treatment group as a covariate and prior warfarin/VKA status (experienced, naïve).
Point estimates and two-sided 95% Cis for HR will be constructed for the outcome.

o Validation of Observational Results Against Aristotle Data
In Objective 1 alone we will validate the findings from our primary analysis against ARISTOTLE by determining whether results of the EHR analysis are compatible with the ARISTOTLE trial results. The ARISTOTLE trial demonstrated superiority of apixaban over warfarin for the primary endpoint (HR 0.79, 95% CI 0.66-0.95). The treatment effect seen with the EHR data may be weaker than that seen in ARISTOTLE.

A subgroup analysis looking at the outcomes of the EU patients in ARISTOTLE showed a smaller treatment difference with the estimate for HR below 1 but upper limit of the CI crossing 1 for the primary efficacy endpoint and death: HR for stroke/SE= 0.92 (95% CI = 0.56; 1.52), HR for all cause death= 0.89 (95% CI = 0.68; 1.18). It was suggested in the European Medicines Agency (EMA) Assessment Report that the smaller treatment effect seen in the EU patients could be due to better INR control in the warfarin arm of the EU subgroup (median TTR 68.93%)[10]. This study could provide additional evidence on this point. Since all the patients in our cohort are from the UK the results can be compared with the results from the ARISTOTLE patients in the EU.

By assessing superiority and non-inferiority we will see whether the treatment effect observed is more similar to the study results as a whole (apixaban superior) or the EU subgroup results. Either a result of superiority or non-inferiority will be considered compatible with the ARISTOTLE trial results. We have set two criteria that must be met for us to conclude results are consistent with the result demonstrated in the trial:
1. The effect size must be clinically comparable with the ARISTOTLE findings; the hazard ratio for time to stroke/systemic embolism with the EHR must be between 0.69 and 0.99. This range is not symmetrical around the ARISTOTLE estimate of 0.79 as it is anticipated that the treatment effect in routine clinical care may be weaker than that seen in the optimised setting of a clinical trial.
2. The upper limit of the 95% confidence interval for the rate ratio must be less than 1.52 (upper limit in the EU subgroup of ARISTOTLE).
In addition, if the upper limit of the 95% CI is less than 1 then superiority of apixaban vs warfarin will be concluded. Either result (superiority or non-inferiority) will be taken as evidence that EHR data may be useful to look at NOACs

Secondary analyses
Secondary outcomes include the key safety outcome of major bleeding (as defined in section N) and the individual outcomes of stroke, systemic embolism, myocardial infarction, and mortality. All secondary outcomes other than major bleeding will use the same analysis approach (primary censoring scheme and regression model) as specified for the primary analysis above. For major bleeding the same regression model as the primary analysis will be used but with the supportive censoring scheme in which patients are censored around the time of last study drug. Within major bleeding results will also be summarised for intracranial, gastrointestinal, and bleeding at other locations. This safety analysis approach will be used for all 3 objectives.

Sensitivity analyses
All primary and secondary efficacy outcomes described above (stroke/SE combined and individually, MI, and mortality) will also be analysed using the supportive censoring scheme described above in which patients are censored around the time of last study drug. This analysis targets the effect of initiation of apixaban vs initiation or continuation of warfarin on the time to event while on prescribed treatment and investigates whether the extent of treatment discontinuation compromises confidence in the primary and secondary efficacy analyses.

The exclusion of patient-time post initially prescribed treatment discontinuation in the safety and sensitivity analyses might bias the results towards a conclusion of no difference[11], for example if those at higher bleeding risk were more likely to discontinue one of the treatment arms due to minor bleeding events than if those same minor

bleeding events occurred with the other treatment. The set of patients who switch or discontinue treatment during the study period will be examined to ascertain whether biases of this nature have occurred. The proportion of patients switching or discontinuing treatment, timings of withdrawal, and their baseline characteristics will be summarised by initially prescribed treatment.

Additional analyses may be performed using methods such as inverse-probability-of-censoring weighting or a rank-preserving structural failure time model to estimate the treatment effect that would have been observed in the absence of treatment switching.

We expect different adherence in routine clinical practice compared with the trial adherence may explain some of the difference in treatment effect observed between routine clinical care and the trial. Adherence will therefore be estimated in the EHR cohort to enable comparisons with the trial and investigate the extent to which this may have influenced differences in treatment effect. With the EHR data we do not know how many tablets a patient has taken or if a given prescription is filled by a patient. We will estimate the proportion of time covered by prescribing as a proxy measure for adherence; this proxy measure assumes that all prescriptions are filled and that a patient takes all tablets in the prescription. This measure of adherence is not expected to accurately estimate the adherence of a given individual but should give an idea of how adherent a patient is compared with others. Prescribing for AF in the UK is predominantly through GPs meaning prescribing information from other potential sources of treatment should not be missing. However, it is possible that a patient's first prescription may be issued in hospital and treatment prescribed during any periods of hospitalisation will not be recorded in a patient's EHR leading to missing exposure data.

We will calculate the proportion of days covered (PDC) over a patient's time when on prescribed treatment as a measure of adherence. PDC will be estimated using patient prescription data including the total number of tablets prescribed, daily dose, and number of days on treatment (derived date of last dose – index date +1). This method of estimating adherence cannot easily be used for warfarin due to daily dose being poorly recorded in EHR; in a sample of CPRD Gold warfarin prescription records many patients had dose recorded with noninformative text such as "take as directed". Depending on the quality of the prescription data warfarin adherence may instead be estimated by looking at patient adherence to other long-term daily medications as a proxy for warfarin adherence; the reliability of this measure would then be explored by comparing this proxy measure in the apixaban users with the PDC calculated directly using the apixaban prescription data.

For warfarin patients INR control will be assessed as a measure of adherence. We will look at INR values to calculate percent Time INR in Therapeutic Range (TTR) where the therapeutic range is 2.0 to 3.0 inclusive. Proportion of time in each INR interval will be calculated using Rosendaal's method. INR is influenced not only by patient adherence to the drug but also by other factors such as diet, alcohol intake, and drug interactions. The ability of a patient to comply with the lifestyle adjustments necessary to maintain INR control on a given warfarin dose may be considered as factors of warfarin treatment adherence. Patient INR control can therefore be used as a measure of overall warfarin treatment regime adherence.

We will perform a supplementary analysis comparing time to event while on treatment in patients deemed to have adequate proportion of time on therapeutic dose (adherent). We will also perform an exploratory subgroup analysis by INR TTR using TTR categories based on the TTR distribution.

P. Plan for addressing confounding

In the EHR cohort study period apixaban was a newly available treatment for the indication of interest leading to the possibility of channelling bias. The analysis cohort in this study is derived from observational data meaning there is likely to be confounding. To handle confounding for all objectives the treatment arms will be matched using the optimal method selected, for example by propensity score matching or coarsened exact matching. For objective 1 by applying the trial inclusion and exclusion criteria to both treatment cohorts and matching using the baseline covariates we should avoid channelling bias. It is possible that unmeasured or unknown confounding may remain and this will be explored and discussed in the analysis of the results.

Q. Plans for addressing missing data

Missing Baseline Data

UK EHR data have been shown to be almost complete for drug prescribing and information on important comorbidity are well recorded. The following variables used for matching may have missing data: weight and BMI, baseline systolic blood pressure, renal function, smoking status, and alcohol intake. Smoking increases the risk of stroke; we therefore anticipate that AF patients are likely to have been asked about their smoking status by their GP. A study comparing performance of ATRIA, CHADS2, and CHA2DS2-VASc risk scores in predicting stroke in patients with AF using CPRD data linked with HES found 6% of patients had smoking status not recorded[12]. Alcohol use can trigger AF symptoms so we also expect that hazardous alcohol intake will be recorded in some cases. Where there are missing data on the baseline characteristics used for matching different approaches will be taken depending on the variable in question. In some cases, such as for renal function and alcohol intake, a patient is more likely to have no data entered if there is no overt clinical evidence of abnormality; in such cases we may take a pragmatic approach and use a simplified version of the variable such as categorising into a binary parameter ("evidence of high alcohol" vs "no evidence of high alcohol intake") with those with no data included in the "no evidence of" groups. For BMI and SBP we cannot assume the data are missing at random as we expect that a patient is less likely to be weighed if they appear to be of healthy weight and is less likely to have blood pressure recorded if they do not have hypertension. Patients with missing BMI or SBP will therefore be excluded from the trial-eligible cohort. The number and proportion of patients with missing data for baseline variables will be summarised and the methods used to deal with the missing data described.

Missing Prescription Data

Treatment may be initiated in secondary care meaning the first prescription of patients newly initiating treatment is missing. To account for these potentially missing first prescriptions we will perform a sensitivity analysis where those newly initiating treatment are assumed to have a missing earlier prescription and therefore assigned an earlier derived index date. Patients who are hospitalised may also have prescriptions issued in secondary care leading to treatment gaps seen in their primary care prescription data. The primary analysis using the Prescribed Population will not be affected by such gaps as patient time is included until the patient is censored or experiences an event regardless of treatment gaps. The supplementary analysis using the On-treatment Population and the safety analysis censor at derived date of last dose and may therefore miss events whilst a patient was on treatment in hospital. We will investigate the occurrence of hospitalisation around treatment discontinuation and assess the potential impact on the results of such missed events by performing a sensitivity analysis with different extended derived dates of last dose (extending the final period to 60 or 90 days after the final prescription estimated end).

Missing Outcome Data

The problem of missing data on the outcome events will be addressed by performing a sensitivity analysis repeating the primary analysis on the ONS and HES-linked cohort. The linked cohort will be assembled by restricting the trial-eligible cohort to the patients with ONS-linked and HES-linked data prior to matching. Occurrence of events and detail of events (such as type of stroke) is expected to be better recorded in the linked cohort.

R. Patient or user group involvement (if applicable)

Patients have not been involved in the setting of this study question. However we will consult with patient groups and relevant charities in communicating the findings of the study.

S. Plans for disseminating and communicating study results, including the presence or absence of any restrictions on the extent and timing of publication

The study protocol will be submitted for publication in BMJ Open. The results of the study will be submitted to peer reviewed journals and will be presented at conferences such as the International Society of Pharmacoepidemiology conference. Results will also be published on the London School of Hygiene and Tropical Medicine website and in the PhD thesis of the principal investigator. Results that may impact on treatment guidelines will be shared with policy makers such as the Medicines and Healthcare products Regulatory Agency and the National Institute for Health and Care Excellence.

Conflict of interest statement:

The principal investigator (PI) is funded by a UK Medical Research Council PhD studentship.

T. Limitations of the study design, data sources, and analytic methods

Some of the criteria that should be assessed for ARISTOTLE eligibility may not be well recorded in CPRD. Criteria such as “increased bleeding risk” are vague and it is not clear exactly which Read codes should be included and time scale considered. Other criteria such as alcohol and drug abuse may not be captured for all patients in CPRD. These limitations are consistent with our aim to select a population as similar as possible to the ARISTOTLE trial population with the acknowledgment that differences will remain. The most important risk factors for the primary outcome of stroke (the components of the CHA2DS2-VASc score for AF stroke risk of age, sex, history of congestive heart failure, hypertension, stroke/TIA history, vascular disease history, and diabetes) are mostly well recorded in CPRD[13].

There are differences in the coding systems used by the two datasets and it is possible that the completeness of coding differs between the two. The potential impact of the different coding systems and completeness of coding will be ascertained by comparisons of the rates of diagnoses, events, baseline variables (such as smoking status, alcohol use, lab values, and vital signs), and prescriptions of interest. Including the data source (Gold or Aurum) as one of the matching variables should prevent discrepancy between the two datasets from biasing the results.

The main focus of the study is the validation of our methodology through assembling a cohort of patients comparable to the patients included in ARISTOTLE and finding similar results to the trial. Criteria to determine the success of the methodology have been pre-specified in the protocol. Given the use of CPRD data to determine treatment effectiveness is not yet well established, a finding that these data are not suitable to answer questions on intended effectiveness will be a useful conclusion.

U. References

1. Granger CB, Alexander JH, McMurray JJV, et al. Apixaban versus Warfarin in Patients with Atrial Fibrillation. N Engl J Med. 2011 Sep 15;365(11):981-992
2. Adderley NJ, Ryan R, Nirantharakumar K, et al. Prevalence and treatment of atrial fibrillation in UK general practice from 2000 to 2016. Heart. 2019;105:27-33.
3. Davis RC, Hobbs FDR, Kenkre JE, et al. Prevalence of atrial fibrillation in the general population and in high-risk groups: the ECHOES study. Europace. 2012;14,1553-1559. doi:10.1093/europace/eus087
4. Hirsh J, Fuster V, Ansell J, Halperin JL. American Heart Association/American College of Cardiology Foundation Guide to Warfarin Therapy. 1 Apr 2003. https://doi.org/10.1161/01.CIR.0000063575.17904.4ECirculation. 2003;107:1692–1711.
5. Ruigómez A, Johansson S, Wallander MA, García Rodríguez LA, Predictors and prognosis of paroxysmal atrial fibrillation in general practice in the UK. BMC Cardiovas Disord. 2005 Jul 11;5:20
6. Iacus SM, King G, Porro G. Causal Inference without Balance Checking: Coarsened Exact Matching. Political Analysis. [Online] Cambridge University Press; 2012;20(1):1–24.
7. Berta P, Bossi M, Verzillo S. % CEM: A SAS Macro to perform Coarsened Exact Matching. J Stat Comput Simul. 2017;87(2):227-238
8. King G, Nielsen R, Coberley C, Pope JE, Wells A. 2011. Comparative Effectiveness of Matching Methods for Causal Inference. Copy at http://j.mp/2nydGlv
9. FDA Providing Regulatory Submissions in Electronic Format - Standardized Study Data: Guidance for Industry (Dec. 2014). https://www.fda.gov/downloads/Drugs/GuidanceComplianceRegulatoryInformation/Guidances/UCM292334.pdf; https://www.cdisc.org/
10. European Medicines Agency. Committee for Medicinal Products for Human Use (CHMP). Assessment report Eliquis apixaban. Procedure No.:EMEA/H/C/002148/X/04/G. 20 September 2012. EMA/641505/2012.
11. Jones B, Jarvis P, Lewis J A, Ebbutt A F. Trials to assess equivalence: the importance of rigorous methods BMJ 1996; 313:36
12. van den Ham HA, Klungel OH, Singer DE, et al. Comparative Performance of ATRIA, CHADS2, and CHA2DS2-VASc Risk Scores Predicting Stroke in Patients With Atrial Fibrillation Results From a National Primary Care Database. J of the American College of Cardiology. 2015 Oct,66 (17):1851-1859.
13. Khan NF, Harrison SE, Rose PW. Validity of diagnostic coding within the General Practice Research Database: a systematic review. Br J Gen Pract 2010; 60 (572): e128-e136. DOI: https://doi.org/10.3399/bjgp10X483562.

List of Appendices

Codelist for Atrial Fibrillation

READ code	READ term
14AN.00	h/o: atrial fibrillation
14AR.00	history of atrial flutter
3272.00	ecg: atrial fibrillation
3273.00	ecg: atrial flutter
G573.00	atrial fibrillation and flutter
G573000	atrial fibrillation
G573100	atrial flutter
G573200	paroxysmal atrial fibrillation
G573300	non-rheumatic atrial fibrillation
G573400	permanent atrial fibrillation
G573500	persistent atrial fibrillation
G573600	paroxysmal atrial flutter
G573z00	atrial fibrillation and flutter nos

<ins>Papers from LSHTM EHR group with outcomes or exposures used in this study</ins>

Warren-Gash, C. Herpes Zoster: Epidemiological Links With Stroke and Myocardial Infarction. J Infect Dis, 2018; 218(suppl_2):S102-S106

Silverwood, R.J. ; Forbes, H.J. ; Abuabara, K. ; Ascott, A. ; Schmidt, M. ; Schmidt, S.A.J. ; Smeeth, L. ; Langan, S.M. Severe and predominantly active atopic eczema in adulthood and long term risk of cardiovascular disease: population based cohort study. BMJ, 2018; 361:k1786

Sinnott, S.J. ; Polinski, J.M. ; Byrne, S. ; Gagne, J.J. Measuring drug exposure: concordance between defined daily dose and days' supply depended on drug class. J Clin Epidemiol, 2016; 69:107-13
