# Supplementary material for: Comparison of oral anticoagulants for stroke prevention in atrial fibrillation using the UK clinical practice research Datalink Aurum: A reference trial (ARISTOTLE) emulation study
Source: PLoS Med. 2024 Aug 29;21(8):e1004377. doi: 10.1371/journal.pmed.1004377 (PMC11361421; doi:10.1371/journal.pmed.1004377)
Supplement: S1 Appendix — Table A1. ARISTOTLE inclusion and exclusion criteria applied to CPRD Aurum. Table A2. Efficacy outcomes results from ARISTOTLE. Table A3. Effectiveness outcomes results in the CPRD Aurum ARISTOTLE-analogous cohort. Table A4. Bleeding outcomes and net clinical outcomes results from ARISTOTLE RCT. Table A5. Bleeding outcomes and net clinical outcomes results in the CPRD Aurum ARISTOTLE-analogous cohort. Table A6. Effectiveness outcomes results in the CPRD Aurum ARISTOTLE-analogous cohort using the on-treatment censoring scheme. Table A7. Treatment status of apixaban and warfarin users in CPRD Aurum ARISTOTLE-analogous cohort during 2.5 years of follow-up. Table A8. Characteristics of apixaban and warfarin users in CPRD Aurum ARISTOTLE-analogous cohort by treatment persistence during 2.5 years of follow-up. Table A9. Effectiveness outcomes results in the CPRD Aurum ARISTOTLE-analogous cohort using later study start date (1 January 2014). Table A10. Summary of noninterventional studies comparing apixaban and warfarin in AF patients. (DOCX) [file pmed.1004377.s003.docx]

**S1 Appendix**

**Table A1: ARISTOTLE Inclusion and Exclusion Criteria Applied to CPRD Aurum**

| **Criteria** | **Implementation Rule and Notes** |
| --- | --- |
| **ARISTOTLE inclusion criteria applied to cohort** | |
| 1. Age ≥ 18 years | Day and month of birth not available therefore calculate age by assuming birthdate=01-July-birthyear. |
| 1. Diagnosis of atrial fibrillation or atrial flutter |  |
| 1. One or more of the following risk factor(s) for stroke: |  |
| 1. Age 75 years or older |  |
| 1. Prior stroke, transient ischemic attack or systemic embolus |  |
| 1. Symptomatic congestive heart failure within 3 months or left ventricular dysfunction with an left ventricular ejection fraction (LVEF) ≤ 40% | If patient has medical record corresponding to congestive heart failure or left ventricular dysfunction diagnosis on or prior to index date. |
| 1. Diabetes mellitus |  |
| 1. Hypertension requiring pharmacological treatment |  |
| **ARISTOTLE Exclusion criteria applied to cohort** | |
| 1. Atrial fibrillation or flutter due to reversible causes (e.g. thyrotoxicosis, pericarditis) |  |
| 1. Clinically significant (moderate or severe) mitral stenosis | Clinical significance not recorded therefore assume if there is a record of mitral stenosis condition is clinically significant. |
| 1. Increased bleeding risk that is believed to be a contraindication to oral anticoagulation (e.g. previous intracranial hemorrhage) |  |
| 1. Conditions other than atrial fibrillation that require chronic anticoagulation (e.g. prosthetic mechanical heart valve) |  |
| 1. Persistent, uncontrolled hypertension (systolic blood pressure > 180 mm Hg, or diastolic blood pressure > 100 mm Hg) | If patient has at least 2 blood pressure readings over the limit in 6 months prior to index date OR code (within 180 days prior to index date) indicating uncontrolled hypertension. |
| 1. Active infective endocarditis |  |
| 1. Required treatment with aspirin > 165 mg/day |  |
| 1. Simultaneous treatment with both aspirin and a thienopyridine (e.g., clopidogrel, ticlopidine) |  |
| 1. Severe comorbid condition with life expectancy of ≤ 1 year |  |
| 1. Active alcohol or drug abuse, or psychosocial reasons that make study participation impractical | Drug or alcohol abuse or any complications of abuse, conditions involving an impaired mental state (dementia including subtypes such as Alzheimer’s), severe mental health conditions (schizophrenia, psychosis, bipolar). |
| 1. Recent ischemic stroke (within 7 days) |  |
| 1. Severe renal insufficiency (serum creatinine > 2.5 mg/dL or a calculated creatinine clearance < 25 mL/min | Lab result of serum creatinine > 2.5 mg/dL or calculated creatinine clearance < 25 mL/min within 90 days prior to index date OR code corresponding to severe renal insufficiency (chronic kidney disease stage 4 or 5, dialysis). |
| 1. ALT or AST > 2X ULN or a Total Bilirubin ≥ 1.5X ULN (unless an alternative causative factor [e.g., Gilbert’s syndrome] is identified) | Lab result showing ALT or AST > 2X ULN or a Total Bilirubin ≥ 1.5X ULN within 90 days prior to index date (AND no diagnosis of Gilbert’s syndrome). |
| 1. Platelet count ≤ 100,000/ mm^3^ | Lab result showing platelet count ≤ 100,000/ mm^3^ within 90 days prior to index date OR a medical record of thrombocytopenia within 90 days prior to index date. |
| 1. Hemoglobin < 9 g/dL | Lab result showing hemoglobin < 9 g/dL within 90 days prior to index date. |
| 1. Inability to comply with INR monitoring | Evidence of drug or alcohol abuse, impaired mental state, severe mental health conditions; excluded by excl criteria number 10 |
| 1. Women of child bearing potential unwilling or unable to use an acceptable method to avoid pregnancy, women who are pregnant or breastfeeding | Exclude women with codes relating to pregnancy, childbirth, antenatal or postnatal care, or breastfeeding in the 3 years prior to index date. |

ALT=alanine aminotransferase; AST=aspartate aminotransferase; CPRD=Clinical Practice Research Datalink; INR=international normalised ratio; ULN = upper limit of normal.
Note: For exclusion numbers 12-15 involving lab results a pragmatic approach was taken in which a patient was assumed not to have the exclusion criteria if there was no lab result available in the 90 days prior to index date and the latest available lab result prior to index date did not meet the criteria.

**Description of modified coarsened exact matching in step 2: selection of apixaban trial-analogous patients:**

A modified form of coarsened exact matching was used in which subgroups of patients were constructed based on sex, age group, prior vitamin K antagonist (VKA) exposure, CHADS_2_ score (stroke risk factor score based on Congestive heart failure, Hypertension, Age ≥ 75 years, Diabetes, prior Stroke), stroke risk factors, and renal function category. Combining the sources of information on the ARISTOTLE patient characteristics allowed us to derive simultaneous equations relating to combinations of these subgroups which could result in a baseline distribution identical to that observed in the apixaban arm of the trial; the equations were then solved numerically giving a range of possible solutions.

Random sampling appropriate numbers of patients from these subgroups resulted in an apixaban ARISTOTLE-analogous cohort of 9,120 patients with similar baseline characteristics to ARISTOTLE participants at the point of randomisation.

**Matching Feasibility**

Ethnicity was limited by the pool of patients available in CPRD. We chose not to match to the trial on concomitant medications as treatment guidelines differ between countries; furthermore since oral anticoagulant (OAC) users in CPRD Aurum were matched on the stroke risk factors which are the indications for these medications, the CPRD Aurum cohort should represent typical prescribing for the trial-analogous cohort in the UK given this baseline distribution of risk factors.

**Description of selection of prevalent users in Step 3: matching of apixaban trial-analogous patients to warfarin trial-eligible patients in CPRD**

Continuing warfarin users in the VKA-experienced strata could be eligible for matching to the switchers to apixaban at multiple different index dates. The method for selection of index date of continuing warfarin users was not specified in the pre-published study protocol to allow testing of prevalent new user design-type methods applied to this data setting and for the objective of trial emulation. The prevalent new user design proposed by Suissa in 2017[1] was designed to avoid the introduction of selection bias when including prevalent users; this method was unsuitable to the objective due to problems with the model convergence and complexity in constructing suitable propensity score models prior to application of the eligibility criteria.

The Webster-Clark method of sampling prevalent users was employed as detailed in Figure 1, with an adaptation to check exclusion criteria at the point of sampling, initially sampling 5 continuing warfarin users per switcher to apixaban before increasing the sample size to 10 continuing warfarin users per switcher. This method has been show in a simulation study to allow inclusion of prevalent users without introducing selection bias (Webster-Clarke 2022 [2]). The adaptation of checking exclusion criteria at the sampling stage and dropping patients from the pool of continuing warfarin users should they not be eligible at the sampled index date serves the purpose of emulating the process of screening into a randomised controlled trial (RCT).

During the sampling procedure switchers from warfarin to apixaban were taken in order of duration of prior VKA treatment history and for each switcher a sample of 10 continuing warfarin users were selected having equivalent prior VKA exposure to the switcher.

**Table A2: Efficacy Outcomes Results from ARISTOTLE**

|  | **Apixaban Group (N=9,120)** | | | **Warfarin Group (N=9,081)** | | |  |
| --- | --- | --- | --- | --- | --- | --- | --- |
| **Outcome** | Patients with Event  *no.* |  | Event  Rate  *%/yr* | Patients with Event  *no.* |  | Event Rate  *%/yr* | Hazard Ratio (95% CI) |
| Primary outcome: stroke or systemic embolism | 212 |  | 1.27 | 265 |  | 1.60 | 0.79 (0.66,0.95) |
| Stroke | 199 |  | 1.19 | 250 |  | 1.51 | 0.79 (0.65,0.95) |
| Ischemic or uncertain type of stroke | 162 |  | 0.97 | 175 |  | 1.05 | 0.92 (0.74,1.13) |
| Hemorrhagic stroke | 40 |  | 0.24 | 78 |  | 0.47 | 0.51 (0.35,0.75) |
| Systemic embolism | 15 |  | 0.09 | 17 |  | 0.10 | 0.87 (0.44,1.75) |
| Key secondary efficacy outcome: death from any cause | 603 |  | 3.52 | 669 |  | 3.94 | 0.89 (0.80,0.998) |
| Other secondary outcomes |  |  |  |  |  |  |  |
| Stroke, systemic embolism, or death from any cause | 752 |  | 4.49 | 837 |  | 5.04 | 0.89 (0.81,0.98) |
| Myocardial infarction | 90 |  | 0.53 | 102 |  | 0.61 | 0.88 (0.66,1.17) |
| Stroke, systemic embolism, myocardial infarction, or death from any cause | 810 |  | 4.85 | 906 |  | 5.49 | 0.88 (0.80,0.97) |
| Pulmonary embolism or deep-vein thrombosis | 7 |  | 0.04 | 9 |  | 0.05 | 0.78 (0.29,2.10) |

CI=confidence interval ; no.=number; yr=year.
ref: C B. Granger et al. Apixaban versus Warfarin in Patients with Atrial Fibrillation. N Engl J Med 2011; 365:981-992, doi: 10.1056/NEJMoa1107039 [3]

**Table A3: Effectiveness Outcomes Results in the CPRD Aurum ARISTOTLE-analogous Cohort**

| **CPRD Aurum ARISTOTLE-analogous Cohort** | **Apixaban Group (N=8,846)** | | | **Warfarin Group (N=8,846)** | | |  |
| --- | --- | --- | --- | --- | --- | --- | --- |
| **Outcome** | Patients with Event *no.* | Person  years | Event Rate *%/yr* | Patients with Event *no.* | Person  years | Event Rate *%/yr* | Hazard Ratio (95% CI) |
| Primary outcome: stroke or systemic embolism | 201 | 15790 | 1.27 | 250 | 19432 | 1.29 | 0.98 (0.82,1.19) |
| Stroke | 173 | 15810 | 1.09 | 216 | 19472 | 1.11 | 0.99 (0.81,1.21) |
| Ischemic or uncertain type of stroke | 145 | 15822 | 0.92 | 157 | 19507 | 0.80 | 1.13 (0.90,1.41) |
| Hemorrhagic stroke | 34 | 15928 | 0.21 | 65 | 19602 | 0.33 | 0.67 (0.44,1.01) |
| Systemic embolism | 30 | 15920 | 0.19 | 35 | 19600 | 0.18 | 1.01 (0.61,1.66) |
| Key secondary outcome: death from any cause | 697 | 15942 | 4.37 | 824 | 19640 | 4.20 | 1.03 (0.93,1.14) |
| Other secondary outcomes |  |  |  |  |  |  |  |
| Stroke, systemic embolism, or death from any cause | 846 | 15790 | 5.36 | 993 | 19432 | 5.11 | 1.04 (0.95,1.14) |
| Myocardial infarction | 125 | 15837 | 0.79 | 150 | 19496 | 0.77 | 1.01 (0.80,1.28) |
| Stroke, systemic embolism, myocardial infarction, or death from any cause | 934 | 15689 | 5.95 | 1091 | 19296 | 5.65 | 1.04 (0.96,1.14) |
| Pulmonary embolism or deep-vein thrombosis | 44 | 15910 | 0.28 | 81 | 19561 | 0.41 | 0.65 (0.45,0.94) |
| **TTR<0.75** | **Apixaban Group (N=4,486)** | | | **Warfarin Group (N= N=4,486)** | | |  |
| **Outcome** | Patients with Event no. | Person  years | Event Rate %/yr | Patients with Event no. | Person  years | Event Rate %/yr | Hazard Ratio  (95% CI) |
| Primary outcome: stroke or systemic embolism | 108 | 7917 | 1.36 | 142 | 9670 | 1.47 | 0.91 (0.73,1.14) |
| Stroke | 90 | 7930 | 1.13 | 122 | 9693 | 1.26 | 0.90 (0.70,1.14) |
| Ischemic or uncertain type of stroke | 75 | 7936 | 0.95 | 91 | 9715 | 0.94 | 1.00 (0.76,1.32) |
| Hemorrhagic stroke | 18 | 7988 | 0.23 | 35 | 9774 | 0.36 | 0.63 (0.38,1.04) |
| Systemic embolism | 18 | 7982 | 0.23 | 21 | 9775 | 0.21 | 1.00 (0.55,1.83) |
| Key secondary outcome: death from any cause | 404 | 7996 | 5.05 | 516 | 9798 | 5.27 | 0.94 (0.84,1.06) |
| Other secondary outcomes |  |  |  |  |  |  |  |
| Stroke, systemic embolism, or death from any cause | 483 | 7917 | 6.10 | 610 | 9670 | 6.31 | 0.95 (0.85,1.06) |
| Myocardial infarction | 70 | 7937 | 0.88 | 95 | 9702 | 0.98 | 0.87 (0.66,1.16) |
| Stroke, systemic embolism, myocardial infarction, or death from any cause | 532 | 7861 | 6.77 | 670 | 9580 | 6.99 | 0.95 (0.85,1.05) |
| Pulmonary embolism or deep-vein thrombosis | 23 | 7979 | 0.29 | 38 | 9757 | 0.39 | 0.73 (0.46,1.16) |

| **TTR≥0.75** | **Apixaban Group (N=4,360)** | | | **Warfarin Group (N=4,360)** | | |  |
| --- | --- | --- | --- | --- | --- | --- | --- |
| **Outcome** | Patients with Event no. | Person  years | Event Rate %/yr | Patients with Event no. | Person  years | Event Rate %/yr | Hazard Ratio  (95% CI) |
| Primary outcome: stroke or systemic embolism | 91 | 7881 | 1.15 | 108 | 9761 | 1.11 | 1.05 (0.82,1.34) |
| Stroke | 80 | 7890 | 1.01 | 94 | 9779 | 0.96 | 1.07 (0.82,1.39) |
| Ischemic or uncertain type of stroke | 67 | 7896 | 0.85 | 66 | 9792 | 0.67 | 1.24 (0.92,1.68) |
| Hemorrhagic stroke | 16 | 7944 | 0.20 | 30 | 9828 | 0.31 | 0.72 (0.43,1.21) |
| Systemic embolism | 12 | 7942 | 0.15 | 14 | 9825 | 0.14 | 0.99 (0.51,1.93) |
| Key secondary outcome: death from any cause | 298 | 7951 | 3.75 | 308 | 9842 | 3.13 | 1.20 (1.04,1.37) |
| Other secondary outcomes |  |  |  |  |  |  |  |
| Stroke, systemic embolism, or death from any cause | 366 | 7881 | 4.64 | 383 | 9761 | 3.92 | 1.19 (1.05,1.34) |
| Myocardial infarction | 53 | 7905 | 0.67 | 55 | 9794 | 0.56 | 1.22 (0.88,1.70) |
| Stroke, systemic embolism, myocardial infarction, or death from any cause | 406 | 7837 | 5.18 | 421 | 9715 | 4.33 | 1.20 (1.06,1.35) |
| Pulmonary embolism or deep-vein thrombosis | 19 | 7938 | 0.24 | 43 | 9803 | 0.44 | 0.54 (0.35,0.84) |

CI=confidence interval; CPRD=Clinical Practice Research Datalink; no.=number; TTR=time in therapeutic range; yr=year.
Note: time to event outcomes analysed using a Cox proportional hazards model with robust standard errors stratified by prior vitamin K antagonist exposure status. Patients were censored at the earliest of (outcome event, death, transfer out of practice, last collection date, 2.5 years after the index date).

For the analysis by TTR inverse probability of treatment weighting was applied to the apixaban users targeting the treatment effect in the warfarin users with TTR <0.75 and TTR ≥0.75.

**Table A4: Bleeding Outcomes and Net Clinical Outcomes Results from ARISTOTLE RCT**

| **ARISTOTLE RCT** | **Apixaban Group (N=9,088)** | | | **Warfarin Group (N=9,052)** | | |  |
| --- | --- | --- | --- | --- | --- | --- | --- |
| **Outcome** | Patients with Event *no.* |  | Event Rate *%/yr* | Patients with Event *no.* |  | Event Rate *%/yr* | Hazard Ratio  (95% CI) |
| Primary safety outcome: ISTH major bleeding | 327 |  | 2.13 | 462 |  | 3.09 | 0.69 (0.60,0.80) |
| Intracranial | 52 |  | 0.33 | 122 |  | 0.80 | 0.42 (0.30,0.58) |
| Other location | 275 |  | 1.79 | 340 |  | 2.27 | 0.79 (0.68,0.93) |
| Gastrointestinal | 105 |  | 0.76 | 119 |  | 0.86 | 0.89 (0.70,1.15) |
| Net clinical outcomes |  |  |  |  |  |  |  |
| Stroke, SE, or major bleeding | 521 |  | 3.17 | 666 |  | 4.11 | 0.77 (0.69,0.86) |
| Stroke, SE, major bleeding, or death from any cause | 1009 |  | 6.13 | 1168 |  | 7.20 | 0.85 (0.78,0.92) |

CI = confidence interval; ISTH=International Society on Thrombosis and Haemostasis; no. = number; SE=systemic embolism; yr = year.

ref: C B. Granger et al. Apixaban versus Warfarin in Patients with Atrial Fibrillation. N Engl J Med 2011; 365:981-992, doi: 10.1056/NEJMoa1107039 [3]

**Table A5: Bleeding Outcomes and Net Clinical Outcomes Results in the CPRD Aurum ARISTOTLE-analogous Cohort**

| **CPRD Aurum ARISTOTLE-analogous Cohort** | **Apixaban Group (N=8,846)** | | | **Warfarin Group (N=8,846)** | | |  | |
| --- | --- | --- | --- | --- | --- | --- | --- | --- |
| **Outcome** | Patients with Event *no.* | Person  years | Event Rate *%/yr* | Patients with Event *no.* | Person  years | Event Rate *%/yr* | Hazard Ratio  (95% CI) | |
| Primary safety outcome: major bleeding | 367 | 14998 | 2.45 | 486 | 17574 | 2.77 | 0.88 (0.77,1.00) | |
| Intracranial | 53 | 15291 | 0.35 | 89 | 17957 | 0.50 | 0.71 (0.51,1.00) | |
| Other location | 91 | 15234 | 0.60 | 114 | 17905 | 0.64 | 0.93 (0.70,1.22) | |
| Gastrointestinal | 230 | 15116 | 1.52 | 302 | 17717 | 1.70 | 0.88 (0.74,1.04) | |
| Net clinical outcomes |  |  |  |  |  |  |  | |
| Stroke, SE, or major bleeding | 514 | 14890 | 3.45 | 631 | 17482 | 3.61 | 0.95 (0.84,1.06) | |
| Stroke, SE, major bleeding, or death from any cause | 1005 | 14890 | 6.75 | 1121 | 17482 | 6.41 | 1.04 (0.96,1.13) | |
| **TTR<0.75** | **Apixaban Group (N=4,486)** | | | **Warfarin Group (N=4,486)** | | |  |  |
| **Outcome** | Patients with Event no. | Person  years | Event Rate %/yr | Patients with Event no. | Person  years | Event Rate %/yr | Hazard Ratio  (95% CI) | |
| Primary safety outcome: major bleeding | 199 | 7489 | 2.66 | 296 | 8353 | 3.54 | 0.74 (0.63,0.86) | |
| Intracranial | 28 | 7644 | 0.37 | 51 | 8605 | 0.59 | 0.62 (0.41,0.92) | |
| Other location | 49 | 7613 | 0.64 | 75 | 8567 | 0.88 | 0.72 (0.52,0.99) | |
| Gastrointestinal | 127 | 7548 | 1.68 | 186 | 8447 | 2.20 | 0.75 (0.61,0.91) | |
| Net clinical outcomes |  |  |  |  |  |  |  | |
| Stroke, SE, or major bleeding | 277 | 7430 | 3.73 | 377 | 8299 | 4.54 | 0.81 (0.70,0.93) | |
| Stroke, SE, major bleeding, or death from any cause | 565 | 7430 | 7.60 | 677 | 8299 | 8.16 | 0.92 (0.83,1.02) | |
| **TTR≥0.75** | **Apixaban Group (N=4,360)** | | | **Warfarin Group (N=4,360)** | | |  |  |
| **Outcome** | Patients with Event no. | Person  years | Event Rate %/yr | Patients with Event no. | Person  years | Event Rate %/yr | Hazard Ratio  (95% CI) |  |
| Primary safety outcome: major bleeding | 166 | 7479 | 2.22 | 190 | 9178 | 2.07 | 1.08 (0.90,1.30) |  |
| Intracranial | 24 | 7616 | 0.32 | 38 | 9304 | 0.41 | 0.80 (0.52,1.24) |  |
| Other location | 42 | 7588 | 0.55 | 39 | 9290 | 0.42 | 1.35 (0.91,1.99) |  |
| Gastrointestinal | 102 | 7538 | 1.35 | 116 | 9225 | 1.26 | 1.07 (0.85,1.35) |  |
| Net clinical outcomes |  |  |  |  |  |  |  |  |
| Stroke, SE, or major bleeding | 232 | 7417 | 3.13 | 254 | 9142 | 2.78 | 1.13 (0.97,1.32) |  |
| Stroke, SE, major bleeding, or death from any cause | 440 | 7417 | 5.93 | 444 | 9142 | 4.86 | 1.22 (1.09,1.37) |  |

CI=confidence interval; CPRD=Clinical Practice Research Datalink; no.=number; SE=systemic embolism; TTR=time in therapeutic range; yr = year.
Note: time to event outcomes analysed using a Cox proportional hazards model with robust standard errors stratified by prior VKA exposure status. Patients were censored at the earliest of (outcome event, death, transfer out of practice, last collection date, derived date of last exposure to index treatment).

For the analysis by TTR inverse probability of treatment weighting was applied to the apixaban users targeting the treatment effect in the warfarin users with TTR <0.75 and TTR ≥0.75.

**Table A6: Effectiveness Outcomes Results in the CPRD Aurum ARISTOTLE-analogous Cohort using the On-treatment Censoring Scheme**

|  | **Apixaban Group (N=8,846)** | | | **Warfarin Group (N=8,846)** | | |  |
| --- | --- | --- | --- | --- | --- | --- | --- |
| **Outcome** | Patients with Event *no.* | Person  years | Event Rate *%/yr* | Patients with Event *no.* | Person  years | Event Rate *%/yr* | Hazard Ratio (95% CI) |
| Primary outcome: stroke or systemic embolism | 196 | 15790 | 1.24 | 230 | 19432 | 1.18 | 1.04 (0.86,1.25) |
| Stroke | 168 | 15810 | 1.06 | 198 | 19472 | 1.02 | 1.04 (0.85,1.27) |
| Ischemic or uncertain type of stroke | 141 | 15822 | 0.89 | 143 | 19507 | 0.73 | 1.19 (0.95,1.50) |
| Hemorrhagic stroke | 32 | 15928 | 0.20 | 61 | 19602 | 0.31 | 0.67 (0.43,1.02) |
| Systemic embolism | 30 | 15920 | 0.19 | 33 | 19600 | 0.17 | 1.07 (0.64,1.76) |
| Key secondary efficacy outcome: death from any cause | 662 | 15942 | 4.15 | 715 | 19640 | 3.64 | 1.12 (1.01,1.25) |
| Other secondary outcomes |  |  |  |  |  |  |  |
| Stroke, systemic embolism, or death from any cause | 809 | 15790 | 5.12 | 877 | 19432 | 4.51 | 1.12 (1.02,1.23) |
| Myocardial infarction | 119 | 15837 | 0.75 | 133 | 19496 | 0.68 | 1.08 (0.84,1.38) |
| Stroke, systemic embolism, myocardial infarction, or death from any cause | 894 | 15689 | 5.70 | 967 | 19296 | 5.01 | 1.12 (1.02,1.22) |
| Pulmonary embolism or deep-vein thrombosis | 43 | 15910 | 0.27 | 73 | 19561 | 0.37 | 0.70 (0.48,1.02) |

CI=confidence interval; CPRD=Clinical Practice Research Datalink; no.=number; yr=year.
Note: time to event outcomes analysed using a Cox proportional hazards model with robust standard errors stratified by prior VKA exposure status. Patients were censored at the earliest of (outcome event, death, transfer out of practice, last collection date, derived date of last exposure to index treatment). These results should be interpreted with caution given evidence of attrition bias in the warfarin arm.

**Table A7: Treatment Status of Apixaban and Warfarin Users in CPRD Aurum ARISTOTLE-analogous Cohort during 2.5 years of Follow-up**

| Subject Disposition  n(%) unless otherwise specified | Apixaban  (N=8 846) |  | Warfarin  (N=8 846) |
| --- | --- | --- | --- |
|  |  |  |  |
| Treatment persistent | 7 785 (88.0) |  | 6 805 (76.9) |
| On treatment until end of 2.5 year follow-up | 3 120 (35.2) |  | 5 191(58.7) |
| On treatment until death | 591 (6.7) |  | 607 (6.9) |
| On treatment until last collection date | 3629 (41.0) |  | 575 (6.5) |
| On treatment until registration end | 445 (5.0) |  | 432 (4.9) |
|  |  |  |  |
| Stopped treatment | 519 (5.9) |  | 596 (6.7) |
| Switched treatment to alternative OAC | 542 (6.1) |  | 1 445 (16.3) |
| Apixaban | N/A |  | 480 (5.4) |
| Warfarin | 149 (1.7) |  | N/A |
| Other VKA | 0 (0.0) |  | 14 (0.2) |
| Dabigatran | 88 (1.0) |  | 156 (1.8) |
| Edoxaban | 94 (1.1) |  | 60 (0.7) |
| Rivaroxaban | 211 (2.4) |  | 735 (8.3) |
|  |  |  |  |
| Time on treatment in months, median (IQR) | 23.2 (12.2,30) |  | 30 (20.0,30) |
| Time to treatment switch in months,  median (IQR) | 7.1 (2.9,15.2) |  | 12.9 (5.8,21.2) |

CPRD=Clinical Practice Research Datalink; IQR=interquartile range; N/A = Not applicable; n=number; OAC=oral anticoagulant; VKA=vitamin K antagonist.
Treatment persistence was ascertained using patient prescription data in CPRD Aurum with change in oral anticoagulant or gaps between prescriptions exceeding 6 months defined as distinct treatment periods.

**Table A8: Characteristics of Apixaban and Warfarin Users in CPRD Aurum ARISTOTLE-analogous Cohort by Treatment Persistence During 2.5 years of Follow-up.**

|  | **Index treatment: Apixaban** | | | **Index treatment: Warfarin** | | |
| --- | --- | --- | --- | --- | --- | --- |
| **Characteristic** | **Apixaban persist (N=7785)** | **Apixaban stop (N=519)** | **Apixaban switch (N=542)** | **Warfarin persist (N=6805)** | **Warfarin stop (N=596)** | **Warfarin switch (N=1445)** |
|  |  |  |  |  |  |  |
| Age – yr, median (IQR) | 71 (63-77) | 67 (57-76) | 72 (63-77) | 71 (64-77) | 66 (57-75) | 70 (62-77) |
| Female sex-no.(%) | 2790 (35.8) | 161 (31.0) | 193 (35.6) | 2455 (36.1) | 182 (30.5) | 553 (38.3) |
| Systolic blood pressure – mm Hg, median (IQR) | 130 (120, 140) | 130 (120, 140) | 130 (120, 140) | 130 (120, 140) | 130 (120, 140) | 132 (120, 140) |
| Weight – kg, median (IQR) | 85 (73, 100) | 85 (73, 99) | 85 (74, 98) | 85 (74, 99) | 85 (74, 99) | 84 (73, 99) |
| Prior myocardial infarction – no. (%) | 964 (12.4) | 52 (10.0) | 74 (13.7) | 833 (12.2) | 63 (10.6) | 178 (12.3) |
| Prior clinically relevant or spontaneous bleeding – no.(%) | 1383 (17.8) | 78 (15.0) | 72 (13.3) | 1142 (16.8) | 103 (17.3) | 262 (18.1) |
| History of fall within previous year – no. (%) | 123 (1.6) | 6 (1.2) | 8 (1.5) | 91 (1.3) | 18 (3.0) | 22 (1.5) |
| Prior use of vitamin K antagonist for >30 consecutive days – no. (%) | 4389 (56.4) | 242 (46.6) | 313 (57.7) | 4060 (59.7) | 230 (38.6) | 654 (45.3) |
|  |  |  |  |  |  |  |
| Qualifying risk factors |  |  |  |  |  |  |
| Age **≥** 75 yr – no. (%) | 2439 (31.3) | 149 (28.7) | 182 (33.6) | 2141 (31.5) | 150 (25.2) | 449 (31.1) |
| Prior stroke, TIA, or systemic embolism – no. (%) | 1537 (19.7) | 73 (14.1) | 101 (18.6) | 1345 (19.8) | 89 (14.9) | 275 (19.0) |
| Heart failure or reduced left ventricular ejection fraction – no. (%) | 2696 (34.6) | 169 (32.6) | 187 (34.5) | 2340 (34.4) | 213 (35.7) | 469 (32.5) |
| Diabetes – no. (%) | 1996 (25.6) | 123 (23.7) | 124 (22.9) | 1779 (26.1) | 138 (23.2) | 358 (24.8) |
| Hypertension requiring treatment – no. (%) | 6752 (86.7) | 441 (85.0) | 469 (86.5) | 5908 (86.8) | 508 (85.2) | 1253 (86.7) |
|  |  |  |  |  |  |  |
| CHADS_2_ score |  |  |  |  |  |  |
| Mean ± SD | 2.1 ± 1.1 | 1.9 ± 1.1 | 2.1 ± 1.1 | 2.1 ± 1.1 | 1.9 ± 1.1 | 2.1 ± 1.1 |
| Distribution – no. (%) |  |  |  |  |  |  |
| 0 | 45 (0.6) | 6 (1.2) | 1 (0.2) | 41 (0.6) | 8 (1.3) | 6 (0.4) |
| 1 | 2552 (32.8) | 221 (42.6) | 198 (36.5) | 2146 (31.5) | 258 (43.3) | 508 (35.2) |
| 2 | 2820 (36.2) | 159 (30.6) | 178 (32.8) | 2554 (37.5) | 173 (29.0) | 512 (35.4) |
| ≥3 | 2368 (30.4) | 133 (25.6) | 165 (30.4) | 2064 (30.3) | 157 (26.3) | 419 (29.0) |
|  |  |  |  |  |  |  |
| Medications at index date – no. (%) |  |  |  |  |  |  |
| ACE inhibitor or ARB | 4931 (63.3) | 265 (51.1) | 333 (61.4) | 4370 (64.2) | 323 (54.2) | 880 (60.9) |
| Amiodarone | 295 (3.8) | 20 (3.9) | 21 (3.9) | 238 (3.5) | 25 (4.2) | 59 (4.1) |
| Beta-blocker | 5388 (69.2) | 342 (65.9) | 353 (65.1) | 4690 (68.9) | 374 (62.8) | 967 (66.9) |
| Aspirin | 440 (5.7) | 37 (7.1) | 37 (6.8) | 413 (6.1) | 49 (8.2) | 95 (6.6) |
| Clopidogrel | 204 (2.6) | 11 (2.1) | 14 (2.6) | 162 (2.4) | 11 (1.8) | 42 (2.9) |
| Digoxin | 1096 (14.1) | 68 (13.1) | 68 (12.5) | 989 (14.5) | 80 (13.4) | 175 (12.1) |
| Calcium blocker | 2650 (34.0) | 148 (28.5) | 167 (30.8) | 2340 (34.4) | 161 (27.0) | 493 (34.1) |
| Statin | 4704 (60.4) | 234 (45.1) | 292 (53.9) | 4141 (60.9) | 291 (48.8) | 796 (55.1) |
| Nonsteriodal antinflammatory agent | 429 (5.5) | 28 (5.4) | 30 (5.5) | 345 (5.1) | 35 (5.9) | 99 (6.9) |
| Gastric antacid drugs | 158 (2.0) | 11 (2.1) | 11 (2.0) | 135 (2.0) | 9 (1.5) | 36 (2.5) |
| Proton pump inhibitor | 2677 (34.4) | 170 (32.8) | 205 (37.8) | 2342 (34.4) | 210 (35.2) | 552 (38.2) |
| H_2_ receptor antagonist | 249 (3.2) | 12 (2.3) | 20 (3.7) | 189 (2.8) | 11 (1.8) | 50 (3.5) |
|  |  |  |  |  |  |  |
| Renal function, creatine clearance – no. (%) |  |  |  |  |  |  |
| Normal, >80 ml/min | 3586 (46.1) | 276 (53.2) | 236 (43.5) | 3076 (45.2) | 304 (51.0) | 694 (48.0) |
| Mild impairment, >50 to 80 ml/min | 2941 (37.8) | 156 (30.1) | 210 (38.7) | 2572 (37.8) | 189 (31.7) | 531 (36.7) |
| Moderate impairment (>30 to 50 ml/min) | 1116 (14.3) | 74 (14.3) | 86 (15.9) | 1026 (15.1) | 89 (14.9) | 191 (13.2) |
| Severe impairment (le 30 ml/min) | 107 (1.4) | 10 (1.9) | 9 (1.7) | 100 (1.5) | 8 (1.3) | 24 (1.7) |
| Not reported | 35 (0.4) | 3 (0.6) | 1 (0.2) | 31 (0.5) | 6 (1.0) | 5 (0.3) |
|  |  |  |  |  |  |  |
| Other risk factors and covariates |  |  |  |  |  |  |
| Peripheral artery disease – no. (%) | 488 (6.3) | 26 (5.0) | 38 (7.0) | 401 (5.9) | 38 (6.4) | 99 (6.9) |
| Aortic plaque – no. (%) | 1846 (23.7) | 112 (21.6) | 139 (25.6) | 1582 (23.2) | 126 (21.1) | 349 (24.2) |
|  |  |  |  |  |  |  |
| Smoking status – no. (%) |  |  |  |  |  |  |
| Non-smoker | 2816 (36.2) | 187 (36.0) | 183 (33.8) | 2461 (36.2) | 205 (34.4) | 498 (34.5) |
| Ex-smoker | 4321 (55.5) | 282 (54.3) | 322 (59.4) | 3801 (55.9) | 322 (54.0) | 822 (56.9) |
| Current smoker | 648 (8.3) | 50 (9.6) | 37 (6.8) | 543 (8.0) | 69 (11.6) | 125 (8.7) |
|  |  |  |  |  |  |  |
| Alcohol consumption – no. (%) |  |  |  |  |  |  |
| Non-drinker | 2449 (31.5) | 169 (32.6) | 184 (33.9) | 2204 (32.4) | 193 (32.4) | 445 (30.8) |
| Light drinker, up to 14 units per week | 3672 (47.2) | 216 (41.6) | 241 (44.5) | 3202 (47.1) | 269 (45.1) | 672 (46.5) |
| Moderate drinker, 15 to 42 units per week | 1366 (17.5) | 105 (20.2) | 92 (17.0) | 1149 (16.9) | 102 (17.1) | 264 (18.3) |
| Heavy drinker, more than 42 units per week | 175 (2.2) | 18 (3.5) | 10 (1.8) | 138 (2.0) | 22 (3.7) | 44 (3.0) |
|  |  |  |  |  |  |  |
| Socioeconomic status – no. (%) |  |  |  |  |  |  |
| England IMD2015 quintile 1(least deprived) | 1974 (25.4) | 138 (26.6) | 134 (24.7) | 1747 (25.7) | 131 (22.0) | 353 (24.4) |
| England IMD2015 quintile 2 | 1842 (23.7) | 122 (23.5) | 134 (24.7) | 1551 (22.8) | 150 (25.2) | 356 (24.6) |
| England IMD2015 quintile 3 | 1509 (19.4) | 88 (17.0) | 118 (21.8) | 1362 (20.0) | 106 (17.8) | 291 (20.1) |
| England IMD2015 quintile 4 | 1261 (16.2) | 94 (18.1) | 88 (16.2) | 1108 (16.3) | 105 (17.6) | 252 (17.4) |
| England IMD2015 quintile 5(most deprived) | 1199 (15.4) | 77 (14.8) | 68 (12.5) | 1037 (15.2) | 104 (17.4) | 193 (13.4) |
|  |  |  |  |  |  |  |
| Ethnicity – no. (%) |  |  |  |  |  |  |
| White | 7421 (95.3) | 488 (94.0) | 515 (95.0) | 6512 (95.7) | 558 (93.6) | 1374 (95.1) |
| Black | 89 (1.1) | 6 (1.2) | 9 (1.7) | 80 (1.2) | 14 (2.3) | 9 (0.6) |
| South Asian | 175 (2.2) | 14 (2.7) | 15 (2.8) | 134 (2.0) | 16 (2.7) | 41 (2.8) |
| East Asian | 9 (0.1) | 1 (0.2) | 0 | 13 (0.2) | 2 (0.3) | 3 (0.2) |
| Mixed | 19 (0.2) | 4 (0.8) | 2 (0.4) | 19 (0.3) | 2 (0.3) | 7 (0.5) |
| Other | 18 (0.2) | 3 (0.6) | 1 (0.2) | 14 (0.2) | 3 (0.5) | 5 (0.3) |
| Unknown | 40 (0.5) | 2 (0.4) | 0 | 23 (0.3) | 1 (0.2) | 1 (0.1) |
|  |  |  |  |  |  |  |
| Charlson comorbidity index components – no. (%) |  |  |  |  |  |  |
| Chronic obstructive pulmonary disease | 990 (12.7) | 71 (13.7) | 77 (14.2) | 847 (12.4) | 81 (13.6) | 213 (14.7) |
| Connective tissue disease | 469 (6.0) | 34 (6.6) | 33 (6.1) | 385 (5.7) | 30 (5.0) | 119 (8.2) |
| Peptic ulcer | 356 (4.6) | 19 (3.7) | 36 (6.6) | 284 (4.2) | 26 (4.4) | 83 (5.7) |
| Liver disease | 61 (0.8) | 5 (1.0) | 10 (1.8) | 44 (0.6) | 7 (1.2) | 10 (0.7) |
| Hemiplegia | 22 (0.3) | 1 (0.2) | 1 (0.2) | 13 (0.2) | 0 | 3 (0.2) |
| Non-haematological Cancer | 956 (12.3) | 54 (10.4) | 56 (10.3) | 881 (12.9) | 84 (14.1) | 181 (12.5) |
| Haematological cancer | 157 (2.0) | 8 (1.5) | 9 (1.7) | 128 (1.9) | 13 (2.2) | 22 (1.5) |
|  |  |  |  |  |  |  |
| BMI – kg/m^2^, median (IQR) | 29 (26, 33) | 29 (25, 33) | 28 (25, 32) | 29 (26, 33) | 28 (25, 33) | 29 (25, 33) |
|  |  |  |  |  |  |  |
| Time in therapeutic range, median (IQR) | N/A | N/A | N/A | 0.78 (0.68, 0.86) | 0.69 (0.50, 0.81) | 0.64 (0.49, 0.78) |

ACE = angiotensin-converting enzyme; ARB = angiotensin-receptor blocker; BMI=body mass index; CHADS_2_=stroke risk factor score based on Congestive heart failure, Hypertension, Age ≥ 75 years, Diabetes, prior Stroke; CPRD=Clinical Practice Research Datalink; IMD2015= Index of Multiple Deprivation 2015; IQR=interquartile range; mo.=number; SD=standard deviation; TIA=transient ischemic attack; yr=year.

Treatment persistence was ascertained using patient prescription data in CPRD Aurum with change in oral anticoagulant or gaps between prescriptions exceeding 6 months defined as distinct treatment periods.
- ‘persist’ patients were those classified as staying on their index treatment during the 2.5 year follow-up period or until censoring
- ‘stop’ patients were those classified as having stopped their index oral anticoagulant treatment without evidence of any subsequent oral anticoagulant exposure in their prescription data during the follow-up period or until censoring.
- ‘switch’ patients were those classified as having switched from their index oral anticoagulant treatment to an alternative oral anticoagulant during the follow-up period or until censoring.

**Table A9: Effectiveness Outcomes Results in the CPRD Aurum ARISTOTLE-analogous Cohort Using Later Study Start Date (01Jan2014)**

| **All Patients** | **Apixaban Group (N=8,753)** | | | **Warfarin Group (N=8,753)** | | |  |
| --- | --- | --- | --- | --- | --- | --- | --- |
| **Outcome** | Patients with Event *no.* | Person  years | Event Rate *%/yr* | Patients with Event *no.* | Person  years | Event Rate *%/yr* | Hazard Ratio (95% CI) |
| Primary outcome: stroke or systemic embolism | 197 | 15667 | 1.26 | 228 | 19291 | 1.18 | 1.06 (0.88, 1.28) |
| Stroke | 171 | 15688 | 1.09 | 199 | 19322 | 1.03 | 1.06 (0.88, 1.28) |
| Ischemic or uncertain type of stroke | 144 | 15697 | 0.92 | 143 | 19351 | 0.74 | 1.23 (0.99, 1.52) |
| Hemorrhagic stroke | 32 | 15805 | 0.20 | 63 | 19435 | 0.32 | 0.65 (0.44, 0.94) |
| Systemic embolism | 29 | 15791 | 0.18 | 32 | 19435 | 0.16 | 1.08 (0.66, 1.77) |
| Key secondary efficacy outcome: death from any cause | 656 | 15815 | 4.15 | 766 | 19466 | 3.94 | 1.05 (0.95, 1.16) |
| Other secondary outcomes |  |  |  |  |  |  |  |
| Stroke, systemic embolism, or death from any cause | 795 | 15667 | 5.07 | 912 | 19291 | 4.73 | 1.07 (0.97, 1.18) |
| Myocardial infarction | 118 | 15724 | 0.75 | 129 | 19341 | 0.67 | 1.13 (0.87, 1.46) |
| Stroke, systemic embolism, myocardial infarction, or death from any cause | 876 | 15578 | 5.62 | 998 | 19173 | 5.21 | 1.07 (0.97, 1.18) |
| Pulmonary embolism or deep-vein thrombosis | 35 | 15787 | 0.22 | 60 | 19414 | 0.31 | 0.71 (0.47, 1.07) |

CI=confidence interval; CPRD=Clinical Practice Research Datalink; no.=number; yr=year.

**Table A10: Summary of Non-interventional Studies Comparing Apixaban and Warfarin in Atrial Fibrillation Patients**

| **Study** | **Description** | **Results** | **Design differences compared to our study** |
| --- | --- | --- | --- |
| Our study | CPRD Aurum linked to HES and ONS, applied trial inclusion/exclusion, matched trial on %VKA-experienced, ITT as primary analysis. | Stroke/SE 0.97 (0.83,1.13)  Ischemic or uncertain stroke 1.11 (0.91,1.35)  ICH 0.64 (0.46,0.89)  All-cause mortality 0.99 (0.91,1.09) |  |
| Vinogradova Y et al 2018 [4] | New users, Qresearch and CPRD Gold linked to HES and ONS, 2011-2016. Censored at treatment stop or switch. Primary prevention study. Apixaban users with AF N=10 601, Warfarin users with AF N=70 585. | Ischemic stroke 1.13 (0.89,1.44)  ICH 0.40 (0.25,0.64)  All-cause mortality 1.31 (1.01,1.25)  Major bleeding 0.66 (0.54,0.79) | In new users alone, did not apply trial criteria or match to the trial, and excluded those with a history of the outcome event for the ischemic stroke and VTE analyses. |
| Larsen TB et al 2016 [5] | Danish databases, IPTW, standard dose apixaban only, ITT. | Ischemic stroke/SE 1.08 (0.91,1.27)  All-cause mortality 0.79 (0.70,0.88) | Danish nationwide databases so may not be as applicable to UK clinical practice, used IPTW to balance covariates, did not match trial baseline characteristics, excluded patients on reduced-dose apixaban, excluded prevalent users, average follow-up 0.9 years in apixaban users. Propensity model did not include as many covariates as ours, therefore possible lower risk of death in apixaban compared with warfarin group may be caused by different baseline risk in warfarin group vs apixaban users. |
| Li XS et al 2017 [6] | US claims, 1:1 PSM, after PSM 38,470 warfarin and 38,470 apixaban, 1 year follow-up, new users, some excl criteria similar to ARISTOTLE,censored at treatment switch or treatment stop+30 days | Stroke/SE 0.67 (0.59,0.76)  Ischemic 0.67 (0.58,0.76) Hemorrhagic 0.70 (0.50,0.99)  SE 0.46 (0.26,0.82) | In new users, in US claims data so may not be as applicable to UK clinical practice, used several of the same criteria as ARISTOTLE and had a large sample size with 38,470 PSM pairs, on-treatment analysis. 9% had CHADS_2_=0 vs 0.6% in trial  11% on amiodarone (similar to trial which had 11% whereas we only had 4%) |
| Proietti et al 2018 [7] | Meta-analysis on real-world use of apixaban for stroke prevention in AF.  Only 1 study from UK  (Lee et al, n=53) | For ‘regular or any dose’ subgroup:  Any thromboembolic event 0.77 (0.64,0.93)  Stroke 0.84 (0.69,1.01)  ICH 0.52 (0.44,0.61)  Major bleeding 0.64 (0.51,0.80) | Systematic review and meta-analysis with only 1 small study contributing UK data. |
| Franklin J et al (protocol) [8] Wang S V et al (results) 2023 [9] | Replication of ARISTOTLE Using US Claims Data  as part of the Emulation of Randomized Clinical Trials with nonrandomized Database Analyses (RCT-DUPLICATE initiative) | Stroke/SE 0.68 (0.61,0.76) | In new users alone whereas our study matches trial in proportion of VKA-experienced users, in US claims data so may not be as applicable to UK clinical practice,  does not match to the trial on baseline characteristics, uses as-treated as primary analysis and ITT secondary analysis, has a shorter follow-up with maximum of 365 days, larger sample size than ours (110,259 matched pairs in protocol) and matches on a wider range of covariates. |

AF=atrial fibrillation; CPRD=Clinical Practice Research Datalink; HES=Hospital Episode Statistics; CHADS2=stroke risk factor score based on Congestive heart failure, Hypertension, Age ≥ 75 years, Diabetes, prior Stroke; ICH=intracranial haemorrhage; IPTW=inverse probability of treatment weighting; ITT=intent to treat; ONS=Office of National Statistics; PSM=propensity score matched/matching; SE=systemic embolism; UK=United Kingdom; US=United States; VKA=vitamin k antagonist; VTE=venous thromboembolism.

**Prediction of TTR in patients on warfarin missing TTR**

To enable inclusion of patients with missing TTR in the analysis by TTR and attempt to minimise the risk of selection bias the data from the patients with TTR data was used to model TTR based on baseline variables (age, sex, BMI, smoking status, diabetes, congestive heart failure, statins, ACEi or ARB, beta-blockers, digoxin, amiodarone, NSAIDs, PPI, prior VKA exposure [naïve, <6 months prior exposure, >= 6 months prior exposure], alcohol consumption, IMD2015_5, renal function, COPD). INR values were restricted to the first year after index date to attempt to minimise selection bias. Two models were trialled for prediction: a mixed model modelling continuous TTR and a logistic regression model modelling TTR categorised as high (TTR >= 0.75) or low (TTR < 0.75) with the model successfully predicting the largest proportion of concordant pairs (observed TTR category vs predicted category) selected. The model was used to predict TTR for the patients on warfarin that were missing TTR thereby allowing all patients to be included in the analysis and attempt to minimise the risk of selection bias.

**Post hoc sensitivity analysis looking at prior INR control**

Prior international normalised ratio (INR) control was not included in the propensity score models for the VKA-experienced due to a high rate of missing prior INR data (missing for 34% in the apixaban arm).

An exploratory post-hoc sensitivity including a prior INR control [categorised as missing INR/poor INR control/good INR control] variable in the propensity score model for the 2 longer prior duration strata was performed. The variable on prior INR control could not be included in the shorter duration strata due to the high rate of missing data (approx. 70% missing in the apixaban shorter duration treatment strata).

Categorisation of INR control was based on the NICE criteria which specifies:

“Reassess anticoagulation for a person with poor anticoagulation control, indicated by any of the following:

- Two INR values higher than 5, or one INR value higher than 8 within the past 6 months.

- Two INR values less than 1.5 within the past 6 months

- Time in therapeutic range (TTR) is less than 65%”

Any patients meeting these NICE criteria based on their INR values in the 6 months prior to their index date were categorised as ‘poor INR control’. Patients with INR values in the 6 months prior to their index date that did not meet these criteria were categorised as ‘good INR control’ and any patients missing or insufficient INR data to determine whether they met the critereia were categorised as ‘missing INR’.

The post-hoc sensitivity analysis including this prior INR control variable in the propensity score models gave results consistent with the primary results [Stroke/SE HR 95%CI 1.02 (0.86,1.21)].

**References**

[1] Suissa S, Moodie EE, Dell'Aniello S. Prevalent new-user cohort designs for comparative drug effect studies by time-conditional propensity scores. Pharmacoepidemiol Drug Saf. 2017 Apr;26(4):459-468. doi: 10.1002/pds.4107.

[2] Webster-Clark M, Mavros P, Garry EM, Stürmer T, Shmuel S, Young J, et al. Alternative analytic and matching approaches for the prevalent new-user design: A simulation study. Pharmacoepidemiol Drug Saf. 2022 Jul;31(7):796-803. doi: 10.1002/pds.5446.

[3] C B. Granger et al. Apixaban versus Warfarin in Patients with Atrial Fibrillation. N Engl J Med 2011; 365:981-992, doi: 10.1056/NEJMoa1107039

[4] Vinogradova Y, Coupland C, Hill T, Hippisley-Cox J. Risks and benefits of direct oral anticoagulants versus warfarin in a real world setting: cohort study in primary care. BMJ. 2018 Jul 4; 362:k2505 doi:10.1136/bmj.k2505. Erratum in: BMJ. 2018 Oct 18;363:k4413.

[5] Larsen TB, Skjøth F, Nielsen PB, Kjældgaard JN, Lip GY. Comparative effectiveness and safety of non-vitamin K antagonist oral anticoagulants and warfarin in patients with atrial fibrillation: propensity weighted nationwide cohort study. BMJ. 2016 Jun 16;353:i3189. doi:10.1136/bmj.i3189.

[6] Li XS, Deitelzweig S, Keshishian A, Hamilton M, Horblyuk R, Gupta K, et al. Effectiveness and safety of apixaban versus warfarin in non-valvular atrial fibrillation patients in “real-world” clinical practice. A propensity-matched analysis of 76,940 patients. Thromb Haemost. 2017 Jun 2;117(6):1072-1082. doi:10.1160/TH17-01-0068

[7] Proietti M, Romanazzi I, Romiti GF, Farcomeni A, Lip GYH. Real-World Use of Apixaban for Stroke Prevention in Atrial Fibrillation: A Systematic Review and Meta-Analysis. Stroke. 2018 Jan;49(1):98-106. doi: 10.1161/STROKEAHA.117.018395.

[8] Franklin J, Brigham and Women's Hospital. Replication of the ARISTOTLE Anticoagulant Trial in Healthcare Claims Data. Available from: <https://www.clinicaltrials.gov/ct2/show/NCT04593030>

[9] Wang SV, Schneeweiss S, RCT-DUPLICATE initiative, Franklin JM, Desai RJ, Feldman W, et al. Emulation of Randomized Clinical Trials With Nonrandomized Databse Analyses: Results of 32 Clinical Trials. JAMA. 2023 Apr 25;329(16):1376-1385. doi:10.1001/jama.2023.4221.
