# Supplementary material for: Comparison of oral anticoagulants for stroke prevention in atrial fibrillation using the UK clinical practice research Datalink Aurum: A reference trial (ARISTOTLE) emulation study
Source: PLoS Med. 2024 Aug 29;21(8):e1004377. doi: 10.1371/journal.pmed.1004377 (PMC11361421; doi:10.1371/journal.pmed.1004377)
Supplement: Attachment — Submitted filename: Responses_to_review.docx [file pmed.1004377.s004.docx]

PLOS Medicine

February 1^st^ 2024

To the Editor,

**RE: Manuscript PMEDICINE-D-23-02702R1response to reviewers’ comments**

Please find as an attachment to this letter an annotated response to reviewers’ comments. We also provide the amended manuscript (one version with changes marked, one clean copy).

In addition to responses to the comments from the editors and reviewers, we have made an additional update to the terminology used to describe the study design in the title and throughout to ensure consistency/standardization with other ongoing trial emulation work. Details are provided at the end of the response document under the heading “Additional updates related to trial emulation terminology”.

Your Sincerely,

Emma Powell
Faculty of Epidemiology and Population Health
London School of Hygiene and Tropical Medicine
Keppel Street, London WC1E 7HT, UK
[maud.teoh@lshtm.ac.uk](mailto:maud.teoh@lshtm.ac.uk)

**Response to review**

We very much appreciate the feedback provided. The original comments are included in the “feedback” column, while full details of our responses and any changes made (or reasons for not making changes) provided in the “Response and details of any changes made” column.

| **#** | **Feedback** | **Response and details of any changes made** |
| --- | --- | --- |
| COMMENTS FROM THE ACADEMIC EDITOR | | |
| 1 | Having said that, there is maybe one limitation that is not addressed in detail, which is the issue of residual confounding. Even properly performed propensity scores cannot take away unmeasured confounding.  The fact that a strong effect is found for death (HR 0.84) for the subjects with TTR in the low range suggests in my opinion that some confounding may still be present, as low TTR is generally associated with worse health and more comorbidity. Also the fact that the effect estimates flip so clearly from below to above one suggests this phenomenon. But even if residual confounding would still be present, I don’t think that would interfere with the conclusion. | Thank you for your comments and for highlighting the issue of residual confounding. A sentence on this important consideration is in the limitations section: ‘There is a risk residual confounding may be present despite the use of propensity score matching’ and we have added this as a main limitation to the abstract: “**The main limitation of the study’s methodology are the risk of residual confounding, channelling bias and attrition bias in the warfarin arm**.”  Thank you for drawing attention to the strong effects seen in the TTR subgroup analysis suggestive of confounding. The analysis method for this subgroup analysis has been improved by applying inverse probability treatment weighting to re-balance the treatment arms on the baseline characteristics within the subgroups. Although the imbalance between the treatment groups within the subgroups was small in the original analysis (which balanced by using the original PS matched pairs) with a maximum mean standardised difference =0.09, it was nonetheless a source of confounding with slight differences in the proportion aged >80, heart failure, and COPD appearing to explain some of the difference in outcomes seen in the original TTR analysis. Removal of this imbalance via IPTW (maximum mean standardised difference = 0.012 across all baseline variables in each subgroup) has brought the subgroup effect estimates closer to the null whilst still showing the same direction of effect and in line with the direction expected (biological plausibility and past studies showing poor INR control is associated with worse outcomes).  This change can be seen in the following locations:  Abstract  “Analysis by TTR subgroup suggested **non-inferiority** of apixaban in those with TTR < 0.75 [Stroke/SE **0.94 (0.75,1.19**), all-cause death **0.99 (0.87,1.12)**]. However, apixaban was associated with increased hazards compared with well-controlled warfarin treatment (TTR ≥ 0.75) [Stroke/SE **1.49 (1.13,1.97)**, all-cause death **1.75 (1.49,2.06**)”  Methods of Analysis  **Supplementary analyses**  **…A subgroup analysis by INR TTR was performed instead** to assess the impact of warfarin control on results **with** all outcomes analysed by TTR-subgroup (TTR < 0.75 and TTR ≥ 0.75). **In order to construct the TTR subgroups whilst maintaining balance in the baseline covariates, inverse probability treatment weighting (IPTW) was used to rebalance the baseline characteristics, applying stabilised weights to the ARISTOTLE-analogous apixaban users. A similar approach to the main analysis was used with propensity score models constructed separately for the new users and warfarin-experienced users.**  Main results – Analysis of impact of warfarin time in therapeutic range (TTR) “TTR was higher in the CPRD cohort than in ARISTOTLE (mean 0.73 vs. 0.62, median 0.76 vs 0.66).  Analysis by TTR subgroup suggested **non-inferiority** of apixaban vs warfarin in those with TTR < 0.75 [Stroke/SE **0.94 (0.75,1.19)**, all-cause death **0.99 (0.87,1.12)**] (Figure 3). Apixaban was associated with increased hazards of both outcomes compared to warfarin in those with well-controlled warfarin treatment (TTR ≥ 0.75) [Stroke/SE **1.49 (1.13,1.97)**, all-cause death **1.75 (1.49,2.06)**] (Figure 3).“  Figure 2 – updated  Safety results  “Analysis by TTR subgroup suggested superiority of apixaban for major bleeding in those with TTR <0.75 [**0.70 (0.59,0.82)**] whereas apixaban users had a higher risk of major bleeding compared with those with optimal warfarin control (TTR ≥ 0.75) [**1.31 (1.08,1.59)**].“  Figure 3 – updated  Discussion paragraph 5  “Our finding that the benefits of apixaban vs warfarin depended on the quality of INR control in the warfarin arm is not unexpected and answers questions raised in the NICE premeeting briefing looking at apixaban in the NVAF population. They noted “the TTR of the trial population receiving warfarin (in ARISTOTLE) may be lower than what is typical in UK clinical practice” and “A lower TTR would be associated with more adverse outcomes in the warfarin arm and apixaban compared with well-controlled warfarin (TTR 75% or more) may not be superior in the long term”.  “ARISTOTLE presented outcomes by centre (for example hospital) TTR which did not show a signal of treatment efficacy of apixaban vs. warfarin differing by centre TTR quartile. We were able to exploit the use of **inverse probability of treatment weighting** to stratify our population based on TTR **whilst maintaining balance in baseline covariates targeting the treatment effect in the different warfarin TTR subgroups**. This showed that in patients well-controlled on warfarin (TTR ≥ 0.75) warfarin is superior to apixaban whereas in patients not well-controlled on warfarin (TTR < 0.75) apixaban is **non-inferior**. These findings may help inform choice of treatment in the context of prior warfarin treatment and availability of INR measurements. “  Limitations of the study design, data sources, and analytic methods  Paragraph 8  “Adherence was difficult to assess due to automatic repeat prescriptions and treatment persistence was more useful in providing a measure of pattern of medicine use over time. In the analysis by TTR subgroup adherence of apixaban users was not accounted for, however, a previous UK study showed apixaban had higher adherence than VKAs [36] meaning we would expect to see better effectiveness outcomes in apixaban; furthermore the use of **IPTW** in the analysis by TTR means predictors of poor adherence are likely to have been balanced between treatments.“ |
| COMMENTS FROM THE EDITORS | | |
| 1 | GENERAL  Please include line numbers starting at 1 on the title page and in continuous sequence throughout, thereafter. | Line numbers added. |
| 2 | GENERAL  Please ensure that the study is reported according to the STROBE guideline, and include the completed STROBE checklist as Supporting Information. Please add the following statement, or similar, to the Methods: "This study is reported as per the Strengthening the Reporting of Observational Studies in Epidemiology (STROBE) guideline (S1 Checklist)." The STROBE guideline can be found here: [http://www.equator-network.org/reporting-guidelines/strobe/](http://protect-eu.mimecast.com/s/LF35CExwoHKOGkXhonyPe?domain=equator-network.org) When completing the checklist, please use section and paragraph numbers, rather than page numbers, as these often change in the event of publication. | STROBE checklist added as supporting information referencing section and paragraph numbers.  Statement added to Methods section. |
| 3 | TITLE In your abstract ‘objective’ you state, ‘To validate non-interventional methodology…’. However, the current title really only reflects the subject matter that you use to ‘showcase’ your methodology. We suggest revising your title to better reflect/include the former, according to PLOS Medicine’s style.   Your title must be nondeclarative and not a question. It should begin with main concept if possible. "Effect of" should be used only if causality can be inferred, i.e., for an RCT. Please place the study design ("A randomized controlled trial," "A retrospective study," "A modelling study," etc.) in the subtitle (ie, after a colon). Perhaps, ‘Comparative analysis of routinely collected data against results from a randomised controlled trial of oral anticoagulants for stroke prevention in atrial fibrillation: A validation study of non-interventional methodology’ or similar? | Thank you for the suggested update to the title, the title of the paper has been updated to the following:  **“Comparison of oral anticoagulants for stroke prevention in atrial fibrillation: a cohort study in the UK Clinical Practice Research Datalink with emulation of a reference trial (ARISTOTLE)”**  This encompasses the points raised, and in addition includes further specifics about the study design and setting of the study that meet STROBE (and RECORD-PE) guidelines, and also clarifies that it is a trial emulation study including the type of trial emulation (see section below “Additional updates related to trial emulation terminology”). |
| 4 | ABSTRACT Please structure your abstract using the PLOS Medicine headings (Background, Methods and Findings, Conclusions). Please combine the Methods and Findings sections into one section, “Methods and findings” Abstract Background: Provide the context of why the study is important. The final sentence should clearly state the study question. | Thank you for highlighting the information missing from the abstract.  The abstract has been updated to reflect PLOS Medicine headings.  Additional information has been added the background to provide context on why the study is important: “Real-world patient data provides an opportunity to evaluate outcomes in a trial analogous population of direct oral anticoagulants (DOACs) users and in patients otherwise excluded from RCTs **however there remains uncertainty on the validity of the methods and suitability of the data**.  **Reference trial emulation involves use of an existing named RCT to (1) inform non-interventional study design and (2) benchmark results against, providing confidence in validity of the selected observational non-interventional methods and data. Methods can then be extended to generate evidence on treatment effects in groups not studied in the original RCT.**  The study question has been added to the end of this section: “**We used linked UK primary care data to investigate whether we could emulate the pivotal ARISTOTLE trial (apixaban vs warfarin) and extend the analysis to investigate the impact of warfarin time in therapeutic range (TTR) on results**.” |
| 5 | Abstract Methods and Findings: Please ensure that all numbers presented in the abstract are present and identical to numbers presented in the main manuscript text. Please include the study design, population and setting, number of participants, years during which the study took place, length of follow up, and main outcome measures. Please define statistical abbreviations at first use for the reader – HR, 95% CI for example. Please quantify the main results with 95% CIs and p values. Please include any important dependent variables that are adjusted for in the analyses. Please include the actual amounts and/or absolute risk(s) of relevant outcomes (including NNT or NNH where appropriate), not just relative risks or correlation coefficients. (example for absolute risks: PMID: 28399126).  In the last sentence of the Abstract Methods and Findings section, please describe the main limitation(s) of the study's methodology. | Abstract numbers cross-checked against main text numbers.  Additional detail on study design added to abstract.  Statistical abbreviation definitions added.  95% Cis used to quantify main results but p values not included (please see reasoning in later section).  Additional detail on the study design added. Actual event rates added for the key outcome.  Sentence on main limitations of the study methodology added: ‘**The main limitations of the study’s methodology are the risk of residual confounding, channelling bias and attrition bias in the warfarin arm**.’ |
| 6 | AUTHOR SUMMARY At this stage, we ask that you include a short, non-technical Author Summary of your research to make findings accessible to a wide audience that includes both scientists and non-scientists. | Author summary added. |
| 7 | METHODS and RESULTS Page 4 – ‘…attack (TIA)/SE…’ please define ‘SE’ here for the reader. Page 5, line 11 – please define ‘PS’ as ‘Propensity Score (PS) model (above table 1 title). Page 5, final paragraph – please revise to ‘threshold of 0.05’ Page 7 para 2 – it might be helpful to redefine ‘SE’ here. | Updated throughout text to add definitions for abbreviations. |
| 8 | Page 7, statistical analysis – thank you for indicating that your study had a prospective protocol/analysis plan. Please include the relevant prospectively written document with your revised manuscript as a Supporting Information file to be published alongside your study, and cite it in the Methods section. A legend for this file should be included at the end of your manuscript. | Protocol to be provided with revised submission.  Sentence added at the beginning of ‘Methods of Analysis’: ‘A prospective protocol was published prior to the analysis detailing the planned analyses [13, also in S2]. ’  Legend added:  ‘**S2: Prospective protocol for the ARISTOTLE emulation study published prior to the analysis**’ |
| 9 | Thank you for detailing the changes made to your analysis in the appendix. Please detail the nature of the changes made to your analysis, including any made in response to peer review, in the Methods section of the paper with rationale. For all observational studies, in the manuscript text, please indicate: (1) the specific hypotheses you intended to test, (2) the analytical methods by which you planned to test them, (3) the analyses you actually performed, and (4) when reported analyses differ from those that were planned, transparent explanations for differences that affect the reliability of the study's results. If a reported analysis was performed based on an interesting but unanticipated pattern in the data, please be clear that the analysis was data-driven. | Thank you for this feedback. The table of changes to the protocol planned analyses has been moved from the appendix to the methods section of the main text.  The table of changes from planned analyses has been updated to ensure all changes from the protocol are present and additional analyses requested by peer review was added.  Additional text has been added to the 'Methods of Analysis' section to clarify hypotheses, planned methods, any changes from the planned methods, and to identify post hoc additional analyses:  **Supplementary analyses**  **A protocol planned analysis in the subset of patients deemed adherent (with adherence measured by TTR in the warfarin users and by proportion of days covered by prescriptions in the apixaban users) was planned to assess the impact of adherence on outcomes. The planned analysis was not possible due to the apixaban prescription data not providing a useful measure of adherence. An analysis by INR TTR was performed instead** ~~T~~to assess the impact of warfarin control on results with all outcomes were also analysed by TTR (TTR < 0.75 and TTR ≥ 0.75). **In order to perform the TTR analysis whilst maintaining balance in the baseline covariates, inverse probability treatment weighting (IPTW) was used to rebalance the baseline characteristics, applying stabilised weights to the ARISTOTLE-analogous apixaban users. A similar approach to the main analysis was used with propensity score models constructed separately for the new users and warfarin-experienced users.**  **An additional post hoc analysis was performed looking at the proportion of apixaban patients prescribed reduced-dose apixaban along with a comparison of the patients meeting the criteria for dose-reduction against the dose actually prescribed**. **In this analysis apixaban dose in the ARISTOTLE-analogous CPRD cohort was assessed and compared against the ARISTOTLE protocol-specified criteria and NICE criteria for reduced apixaban dose. ARISTOTLE specified that participants meeting any 2 of the following criteria assessed at the time of randomisation should have their apixaban dose reduced to 2.5mg BID: age ≥ 80 years, body weight ≤ 60 kg, or serum creatinine ≥ 1.5 mg/dL. These criteria are equivalent to the NICE guidelines for dose reduction with NICE having an additional criteria indicating reduced dose in those with creatinine clearance 15–29 mL/minute.**  **In addition, to assess the impact of the quality of dose-adjustment in the CPRD cohort on the observed effectiveness of apixaban relative to warfarin, a supplementary post hoc analysis was performed looking at the results in the subset of apixaban patients prescribed the correct dose compared with IPTW re-balanced warfarin comparators.**  ~~Validation~~**Benchmarking** of results against ARISTOTLE  **The study hypothesis was that results in the CPRD ARISTOTLE-analogous cohort would be comparable to the ARISTOTLE results, as defined by the pre-specified benchmarking criteria. A slightly weaker benefit of apixaban vs warfarin was expected based on the weaker benefit seen in the EU subgroup of ARISTOTLE and an expectation that the quality of warfarin control in UK patients may be higher than that observed in ARISTOTLE.**  The ~~validation~~ **benchmarking** criteria for considering the results **in the trial-analogous CPRD cohort** to be comparable with ARISTOTLE were pre-specified and published previously [14]:  1. **The effect size must be clinically comparable with the ARISTOTLE findings; the HR for time to stroke/SE with the HR must be between 0.69 and 0.99. This range is not symmetrical around the ARISTOTLE estimate of 0.79 as it is anticipated the treatment effect in routine clinical care may be weaker than that seen in the optimised setting of a clinical trial.**  **2. The upper limit of the 95% CI for the HR for time to stroke/SE must be less than 1.38 (non-inferiority margin used in ARISTOTLE, updated since protocol – see Table 2).**  **The benchmarking step applied only to the primary effectiveness outcome in the trial-analogous CPRD cohort; results in other groups such as patients underrepresented or excluded from the trial would not be expected to remain consistent to the RCT results given the relative risks may differ in these groups. Comparability of other outcomes was to be assessed descriptively with no formal criteria or hypothesis testing used.** |
| 10 | We appreciate your effort to ensure that your manuscript is succinct, but the methods section is currently rather brief, relying on the reader to refer to different pieces of documentation for nuance. Please provide additional detail of the databases you use and of the ARISTOTLE trial itself in the main manuscript. | Thank you for noting the lack of detail that remained in the manuscript. Information on the databases used and on ARISTOLTE has been added to the main manuscript. Additional detail on the methods used has been provided, in particular on the method for inclusion of prevalent users and on the validation criteria to avoid readers having to refer to the protocol:  Settings/data sources  **UK Electronic Healthcare Records**  This study used non-interventional data from UK Clinical Practice Research Datalink (CPRD) Aurum**, a database containing anonymised data from 738 primary care practices across England (approximately 13% of the population of England with 19 million patient records and 7 million active as of September 2018 [14]. CPRD Aurum contains information on clinical diagnoses, prescribing, referrals, tests and demographic/lifestyle factors and is representative of the population of England in geographical spread, social deprivation, age and sex [14]. This study also used 2 additional data sources linked to CPRD Aurum**: Hospital Episodes Statistics (HES) data, **which contains data on patients admitted to NHS hopsitals including diagnoses, admission and discharge,** and Office of National Statistics (ONS) mortality data.  **The reference trial (ARISTOTLE)**  **ARISTOTLE was a randomised, double-blind trial completed in 2011, comparing apixaban with warfarin in the prevention of stroke and SE. The trial included 18201 patients with AF and at least one additional risk factor for stroke. The trial was designed to test for non-inferiority of apixaban compared with warfarin (non-inferiorirty margin of 1.38 for the upper limit of the 95% CI of the hazard ratio for the primary outcome), and showed apixaban superiority for (1) the primary outcome of stroke or SE (HR 0.79; 95% CI 0.66, 0.95),7 (2) the safety endpoint of major bleeding (HR 0.69; 95% CI 0.60, 0.80), and (3) death from any cause (HR 0.89; 95% CI 0.80, 0.99). The ARISTOTLE findings led to the National Institute for Health and Care Excellence (NICE) guidelines on stroke prophylaxis in patients with AF recommending apixaban as a treatment.**  **ARISTOTLE eligibility criteria and** summary baseline patient characteristics were used **to select a cohort of patients from CPRD Aurum analogous to the ARISTOTLE participants.**  The use of CPRD and ARISTOTLE are described in a previous publication [13, also in Appendix] and use of CPRD for this project was approved by the MHRA Independent Scientific Advisory Committee. All data used in this study were anonymised.  Patient Selection  Step 3: matching of apixaban trial-analogous patients to warfarin trial-eligible patients in CPRD  **To emulate ARISTOTLE which stratified randomisation on prior VKA exposure status, patients in the CPRD cohort were matched separately within the VKA-naïve and VKA-experienced strata. A 3-step procedure, based on methods proposed by Suissa et al [20] and Webster Clark et al [21], was used to select and match patients in the VKA-experienced strata whilst avoiding selection bias; this procedure is sumamrised in Figure 1 and described in the Appendix.**  The trial-analogous CPRD Aurum apixaban patients were matched to warfarin CPRD Aurum patients **using greedy nearest-neighbour matching on the logit of the propensity score (PS); a** caliper **of** 0.2 **times the standard deviation of the logit of the propensity score was used for matching as recommended by Austin [21].**  **Validation criteria** text added – provided in response to point 9 above**.**  **S3 Appendix**  Step 3: matching of apixaban trial-analogous patients to warfarin trial-eligible patients in CPRD  **Continuing warfarin users in the VKA-experienced strata could be eligible for matching to the switchers to apixaban at multiple different index dates. The method for selection of index date of continuing warfarin users was not specified in the pre-published study protocol to allow testing of prevalent new user design-type methods applied to this data setting and for the objective of trial emulation. The prevalent new user design proposed by Suissa [19] was designed to avoid the introduction of selection bias when including prevalent users; this method was unsuitable to the objective due to problems with the model convergence and complexity in constructing suitable propensity score models prior to application of the eligibility criteria.**  **The Webster-Clark method of sampling prevalent users [20] was employed as detailed in Figure 1, with an adaptation to check exclusion criteria at the point of sampling, initially sampling 5 continuing warfarin users per switcher to apixaban before increasing the sample size to 10 continuing warfarin users per switcher. This method has been shown in a simulation study to allow inclusion of prevalent users without introducing selection bias [20]. The adaptation of checking**  **exclusion criteria at the sampling stage and dropping patients from the pool of continuing warfarin users should they not be eligible at the sampled index date serves the purpose of emulating the process of screening into an RCT.**  **During the sampling procedure switchers from warfarin to apixaban were taken in order of duration of prior VKA treatment history and for each switcher a sample of 10 continuing warfarin users were selected having equivalent prior VKA exposure to the switcher.** |
| 11 | Throughout the main manuscript where you report statistical information, please ensure to define all statistical abbreviations for the reader at first use. When reporting 95% CIs please use commas to separate upper and lower bounds as hyphens can be confused with reporting of negative values. Please also report p values where reporting 95% CIs.  Page 8, para 2 – please quantify percentages with numerators and denominators. For example, page 10, para 3 should read– ‘[HR 0.79 (95% CI 0.66,0.95); p</=]’. Please check and amend throughout all sections of the main manuscript and supporting files to ensure accuracy in reporting. Note that later you also use the word ‘to’. | All 95% CIs updated to use commas to separate the bounds.  Thank you for highlighting the question surrounding p values. P values are not reported as the focus of the pre-specified validation criteria and comparison of cohort results against the trial results was on the point estimates and the CI boundaries. Furthermore the design of the RCT being emulated focused on the upper limit of the 95% CI, ARISTOTLE SAP text: “the NI of apixaban relative to warfarin will be demonstrated if the upper bound of the two-sided 95% CI for RR is less than 1.38;”.  A p value in this study could come from a test for non-inferiority or superiority (ARISTOTLE planned to test for NI first followed by superiority for the primary efficacy endpoint).  Should a p value be included for this cohort then it would relate to this NI margin for the primary outcome however this is more intuitively understood by a reader comparing the upper limit of the CI to the NI margin.  In addition, HRs are presented for multiple outcomes yet no formal hierarchy or adjustment methods for multiple testing was pre-specified. Inclusion of p values without appropriate adjustment for multiple testing or following a hierarchy could be misleading to the reader. |
| 12 | Page 10, final para – please use an alternative to ‘…AF patients…’ perhaps, ‘…patients with AF on…’ or similar. Please check and amend throughout all subsections of the manuscript, tables, figures and supporting files where relevant. | Updated throughout text to use ‘patients with AF’. |
| 13 | TABLES Throughout when reporting p values please report as <0.001 and where higher the exact p value as 0.002, for example. Please amend throughout all sections of the main manuscript and appendix where relevant. Throughout, when using abbreviations please ensure that all are clearly defined for the reader in an appropriate footnote/caption, including those used for statistical reporting. For example, table 1 – AF, CHADS2, TIA and others are outstanding and table 2 – CI, TTR. Please check and amend throughout all tables in the main manuscript and supporting information. | All tables checked to add all definitions used in the footnotes. |
| 14 | FIGURES Throughout, please ensure to define all abbreviations within an appropriate footnote, including those used for statistical reporting. For example, figure 1, ‘VKA, RCT, PSM’ and ‘figure 2, ‘CPRD, TTR’. Throughout, when reporting 95% CIs please use commas instead of hyphens to separate upper and lower bounds as the latter can be confused with reporting of negative values. Throughout, where 95% CIs are reported please also report p values. As above, please report p as <0.001 and where higher the exact p value as 0.002, for example. Please check and amend throughout all sections of the main manuscript and supporting files. Please ensure your clearly define (in the figure captions) the meaning of the dots and lines used in the forest plots. | I apologise that these abbreviations were missed, I have checked through the figures and added to the footnotes. Figures updated to remove use of non-standard abbreviations where possible.  In addition on review I noticed the dashed line showing the non-inferiority margin should not have been included in Figure 3 showing safety outcomes as this margin is relevant to the primary effectiveness outcome only.  95% CIs have been updated throughout to use commas. Interquartile range have also been updated to use comma separation in the table.  Captions updated to provide additional information on dots and lines, key information on the method, abbreviations. |
| 15 | DISCUSSION Thank you for structuring your discussion according to PLOS Medicine’s style. Please remove all sub-headings from the discussion such that it reads as continuous prose. The opening sentence of the discussion is very long and as such rather inaccessible to the reader. Please revise for brevity and clarity summarizing the main findings of your study. It would be helpful to clearly and explicitly state how and where your data outcomes differ and align with those of the ARISTOTLE trial.  Please remove the conflict of interest, funding and data availability statements from the end of the main manuscript and include only in the manuscript submission form. In the event of publication, they will be compiled as metadata. | Subheadings removed from discussion section.  Thank you for noticing the lack of clarity in the opening sentence; this text has been revised to split into shorter more readable sentences:  In our ARISTOTLE-analogous CPRD Aurum cohort we found results ~~consistent with the trial results~~ that met our predefined criteria for comparability with ARISTOTLE**. We saw non-inferiority of apixaban vs warfarin for time to stroke or systemic embolism but did not see superiority of apixaban vs warfarin as was seen in ARISTOTLE.** ~~, with t~~**T**he ~~slight~~ differences in overall treatment-effect estimates ~~appearing to be~~ **may be** explained by **the lower proportion of Asian patients in our cohort, and** differences in INR control in the warfarin arm of our cohort compared with the ARISTOTLE participants randomised to warfarin.  Conflict of interest, funding and data availability statements have been removed from main manuscript. |
| 16 | SUPPORTING INFORMATION Please ensure that all tables and figures in the supporting information follow the same guidance as detailed above for the main manuscript. Please cite your Supporting Information as outlined here: [https://journals.plos.org/plosmedicine/s/supporting-information](https://protect-eu.mimecast.com/s/IoajCJQBwUPlzgMf9SrWO?domain=journals.plos.org) | Supporting information files S1, S2, S3 provided. Captions added at the end of the manuscript:  **S1: STROBE checklist**  **S2: Prospective protocol for the ARISTOTLE emulation study published prior to the analysis.**  **S3: Appendix containing supporting information.** |
| 17 | REFERENCES In the bibliography, please ensure that you list up to but no more than 6 author names followed by et al. For all web references please ensure you include an, ‘Accessed [date].’ Journal name abbreviations should be those listed in the National Center for Biotechnology Information (NCBI) databases. | References have been updated. |
| **Reviewer #1:** | | |
| 1 | Thank your your hard work. It's well-written and well-cited report. However, I am not convinced that these analyses add anything new. The subanalysis of "special" groups e.g. patients with cancer, obesity etc. may be more interesting as data are limited. | Thank you for your comment; the focus of this paper is on the methodology of RCT emulation and inclusion of prevalent users in this therapeutic area and resulting validation of the use of these non-interventional data sources for answering questions on drug effectiveness, the comparison of results in the cohort against the RCT and the impact of TTR. We acknowledge the analysis of special groups will provide more novel results from a clinical perspective and is work that we plan to do when we extend the methods used in this paper. |
| 2 | There are known racial differences in the treatment effect of OACs. In your study, 95.7% of patients were White, but in the Aristotle 14.4% of patients were Asians. My concern is the generalizability of the study; and it is worth to discuss as a study limitation. | Thank you for raising this important difference between the CPRD cohort and the RCT population, a paragraph has been added to the limitations section:  “**A key limitation of the study was not being able to match ARISOTLE on ethnicity as the CPRD Aurum cohort included a low number of patients from Asian, Black, and Hispanic groups when compared with the RCT (ARISTOTLE had 14.5% participants Asian vs 2.4% in the ARISTOTLE-analogous CPRD cohort). Even in the full ARISTOTLE-eligible CPRD Aurum cohort only 774 of the patients prescribed apixaban in the study period were Asian. There are known racial differences in the treatment effects of OACs with Asian patients experiencing a higher risk of haemorrhagic stroke and intracranial haemorrhage compared with white patients; in ARISTOTLE Asian participants experienced double the risk of stroke or systemic embolism when on warfarin therapy when compared with white participants: ARISTOTLE: RR 2.02, 95% CI 1.52–2.66 [35]. The reasons for the increased risk in Asian patients is not fully understood but is hypothesised to be associated with an increased prevalence of cerebral microbleeds. The difference in proportion of Asian patients between the CPRD cohort and ARISTOTLE is therefore likely to explain some of the difference in treatment effect observed and limits the generalisability of the study with the results of this study relevant to white patients**.” |
| 3 | Do you have the data on Apixaban doses? Were they well-adjusted. It is also worth mentioning the higher number of patients with CKD (comparing to the Aristotle), which may affect dosing. | We do have data on Apixaban dose, I have added a section to results ‘Analysis of quality of apixaban dose-adjustment’ covering the numbers on each dose as well as the numbers meeting the criteria for dose reduction.  In the Methods of Analysis section: “**An additional post hoc analysis was performed looking at the proportion of apixaban patients prescribed reduced-dose apixaban along with a comparison of the patients meeting the criteria for dose-reduction against the dose actually prescribed. In this analysis apixaban dose in the ARISTOTLE-analogous CPRD cohort was assessed and compared against the ARISTOTLE protocol-specified criteria and NICE criteria for reduced apixaban dose. ARISTOTLE specified that participants meeting any 2 of the following criteria assessed at the time of randomisation should have their apixaban dose reduced to 2.5mg BID: age ≥ 80 years, body weight ≤ 60 kg, or serum creatinine ≥ 1.5 mg/dL. These criteria are equivalent to the NICE guidelines for dose reduction with NICE having an additional criteria indicating reduced dose in those with creatinine clearance 15–29 mL/minute.**  **In addition, to assess the impact of the quality of dose-adjustment in the CPRD cohort on the observed effectiveness of apixaban relative to warfarin, a supplementary post hoc analysis was performed looking at the results in the subset of apixaban patients prescribed the correct dose compared with IPTW re-balanced warfarin comparators**.”  **Analysis of apixaban dose-adjustment**  **The proportion of patients meeting the criteria for reduced dose apixaban was similar between the CPRD ARISTOTLE-analogous apixaban, warfarin, and RCT apixaban groups (4.9%, 4.9%, and 4.7% respectively). When including the additional NICE criteria of creatinine clearance 5.1% of apixaban users in the ARISTOTLE-analogous cohort had an indication for reduced-dose apixaban yet a larger proportion (14.3%) were prescribed reduced dose apixaban implying some patients in CPRD Aurum may have been prescribed the wrong dose and/or information on criteria for dose reduction may have been missing from CPRD Aurum.**  **Table 4 added**  **A futher analysis of the quality of dose-adjustment in patients in CPRD Aurum indicated 10.5% of patients may have been prescribed an incorrect dose of apixaban at the index prescription based on the data contained in their EHRs. The majority of incorrect dose relating to patients being prescribed reduced-dose apixaban despite not meeting the criteria for dose reduction. A large proportion of patients prescribed an incorrect dose had only 1 dose adjustment criteria (59.6% of those with incorrect dose) suggesting some prescribers may have thought a dose reduction was warranted when only 1 criteria was present. Other possible reasons for the incorrect dose-adjustment observed here may be data on the criteria missing from the EHR record (ie incorrect ascertainment) or consideration of other medical history which made a prescriber adjust the dose.**  **Table 5 added**  **To assess the impact of the quality of dose-adjustment in the CPRD cohort on the effectiveness of apixaban a supplementary post hoc anlaysis was performed looking at the results in the subset of apixaban patients prescribed the correct dose (N=7921) compared with IPTW re-balanced warfarin comparators. The results in this subset were consistent with the primary results showing apixaban to be non-inferior to warfarin (Stroke/SE 0.96 [0.78,1.17], death 0.97 [0.87,1.09]) with the results moving slightly closer to those observed in ARISTOTLE.**  In the Discussion:  Paragraph 1: “**The higher proportion of apixaban participants prescribed the lower dose of apixaban compared with ARISTOTLE may have contributed to the weaker benefit of apixaban over warfarin observed. We saw evidence suggesting sub-optimal dosing of apixban in some patients in the CPRD cohort with low-dose apixban prescribed to patients without evidence of meeting the criteria for the lower dose.”** |
| **Reviewer #2: Alex McConnachie, Statistical Review** | | |
|  | Subgroup analyses are done by TTR. Is this just for the patients who switched from warfarin to apixaban, with TTR relating to the period prior to the index date? I note that TTR is not included in Table 2. Also, TTR does not appear to be included in Table 1, as one of the covariates used in the PS matching procedure - should it be? If channelling is a risk, then surely patients with worse TTR might be more likely to be switched to apixaban? | We apologise this was not clearer in the original text, you are correct that the subgroup analysis was performed in relation to TTR of warfarin users after the index date as opposed to TTR prior to index date. This means the analysis by TTR included both new users of warfarin or apixaban (VKA-naïve) as well as those that switched from warfarin to apixaban or continued on warfarin (VKA-experienced).  Thank you for highlighting the risk of channelling bias relating to prior TTR. A sentence in the limitations section has been added: “**The availability of new alternative treatments during the study period also means there is a risk of channelling bias in that over time the patients still on warfarin are more likely to be those doing well on warfarin. INR control prior to the index date was not included in the propensity score for the prevalent users due to a high rate of missing data, however, other variables associated with poor INR control were included in the models and an exploratory post-hoc analysis including a variable for poor INR control gave results consistent with the primary results**.”  Channelling bias is also mentioned in a sentence added to the abstract on the main study limitations: “**The main limitation of the study’s methodology is the risk of residual confounding and both channelling bias and attrition bias in the warfarin arm**”.  TTR was not included in the PS matching procedure due to a high rate of missing prior INR data (missing for 34% in the VKA-experienced apixaban arm). The fact that multiple variables associated with poor INR control such as age, weight, and concomitant medications are included in the PS model should help mitigate against not being able to include this variable.  An exploratory post-hoc sensitivity including a prior INR control [categorised as missing INR/poor INR control/good INR control] variable in the PSM for the 2 longer prior duration strata gives results consistent with the primary results [Stroke/SE HR 95%CI 1.02 (0.86,1.21)]. The variable cannot be included in the shorter duration strata due to the high rate of missing data (approx. 70% missing in the apixaban shorter duration trt strata). This categorisation of INR control was based on the NICE criteria which specifies: “  Reassess anticoagulation for a person with poor anticoagulation control, indicated by any of the following:   - Two INR values higher than 5, or one INR value higher than 8 within the past 6 months. - Two INR values less than 1.5 within the past 6 months - Time in therapeutic range (TTR) is less than 65%”     Details of this post hoc sensitivity analysis are included in the manuscript and appendix:  “**Prior INR control was not included in the propensity score models for the VKA-experienced due to a high rate of missing prior INR data (missing for 34% in the apixaban arm). A post-hoc sensitivity analysis including a prior INR control variable in the PSM gave results consistent with the primary results [Stroke/SE HR 95%CI 1.02 (0.86,1.21)]. Details of this post hoc analysis are in S3 Appendix**.” |
|  | Another possibility is that the subgroup analysis is performed in relation to the TTR of the warfarin users after the index date. After multiple reads of the paper, I believe this may be what was done. Is it appropriate to stratify by a post-baseline characteristic? | Thank you for raising this important point.  The subgroup analysis was indeed performed in relation to TTR of warfarin users after the index date. The analysis of outcomes by TTR subgroup was performed for the full cohort including both newly initiating warfarin and those continuing on warfarin and was based on TTR during the study period (from index date onwards) as opposed to TTR prior to index date.  It is generally not recommended to stratify by a post-baseline characteristic (improper subgroup) and a sentence has been added on this point in the limitations section:  “**The analysis of TTR is limited by this being a post-baseline measure available for only one treatment arm; this limitation was also evident in the RCTs of DOACs vs warfarin**.”  However, it is not possible to answer questions on the impact of TTR on outcomes (which is of interest to clinicians) without using a post-baseline measure. ARISTOTLE and other similar RCTs of DOAC vs warfarin performed analyses on outcomes in different TTR strata based on TTR measured post randomisation, and our analyses are therefore analogous to ARISTOTLE. ARISTOTLE analysis was completed at a time when propensity score based methods were not commonly used in RCTs; the FDA statistical review noted that for the purpose of assessing impact of TTR matching the trial participants was not possible. We may conjecture whether methods such as IPTW re-balancing would be considered for exploring the impact of TTR if ARISTOTLE and similar RCTs were conducted more recently now that these propensity score methods are beginning to be adopted for certain contexts in RCTs.  We updated the analysis method to use IPTW as opposed to the use of PS-matched pairings in the original analysis to improve the balance of baseline variables in the TTR-groups. This update has removed a slight imbalance that was causing confounding. In the updated analysis the subgroups are well balanced (all variables in the baseline table have standardised difference <=0.012) with the results brought closer to the null. |
|  | For any subgroup analysis I would normally ask to see an interaction test p-value, but that may not be appropriate here (or necessary, given the magnitude of the difference in treatment effect estimates). | Thank you for raising this point; given the nature of this analysis (improper subgroup with no common variable across the two treatment arms) it was not possible to assess this variable via an interaction test which is the optimal method normally used. The RCTs in this area suffered the same limitation with ARISTOTLE grouping participants based on site TTR quartile and assessing the impact of TTR by comparing the treatment effect across the different TTR quartiles. This method has its own limitations given that quality of warfarin control may be related to individual patient factors and not solely site level quality of care. I have added text to draw attention to looking at results stratified by a post-baseline measure as a limitation. The aim of this analysis was to help answer the question posed in the NICE review on whether apixaban was superior to well-controlled warfarin (TTR > 0.75).  The method for the TTR analysis has also been improved by applying IPTW to remove imbalance (re-weighting the apixaban patients to match the warfarin groups defined by TTR). The new estimates show a similar overall trend but with the removal of the confounding bringing the estimates closer to the null.  Furthermore to remove ambiguity on this point we have removed reference to ‘subgroup’ and instead refer to this as analysis by TTR. This analysis with the method of IPTW targeting the effect in the warfarin users defined by TTR is likely better described avoiding the term ‘subgroup’ which implies grouping by a variable that can be measured in both treatment groups.  These updates can be seen in the manuscript:  Abstract  “Analysis by TTR ~~subgroup~~ suggested ~~superiority~~ **non-inferiority** of apixaban in those with TTR < 0.75 [Stroke/SE ~~0.88 (0.67 to 1.17)~~ **0.94 (0.75,1.19)**, all-cause death ~~0.84 (0.73 to 0.98)~~ **0.99 (0.87,1.12)**]. However, apixaban was associated with increased hazards compared with well-controlled warfarin treatment (TTR ≥ 0.75) [Stroke/SE ~~1.86 (1.38 to 2.52)~~ **1.49 (1.13,1.97)**, all-cause death ~~2.18 (1.84 to 2.60)~~ **1.75 (1.49,2.06)**].”  **Supplementary analyses**  **A protocol planned analysis in the subset of patients deemed adherent (with adherence measured by TTR in the warfarin users and by proportion of days covered by prescriptions in the apixaban users) was planned to assess the impact of adherence on outcomes. The planned analysis was not possible due to the apixaban prescription data not providing a useful measure of adherence. An analysis by INR TTR was performed instead** ~~T~~to assess the impact of warfarin control on results **with all outcomes** ~~were also~~ **analysed by TTR** ~~subgroup~~ (TTR < 0.75 and TTR ≥ 0.75)**. In order to perform the TTR analysis whilst maintaining balance in the baseline covariates, inverse probability treatment weighting (IPTW) was used to rebalance the baseline characteristics, applying stabilised weights to the ARISTOTLE-analogous apixaban users. A similar approach to the main analysis was used with propensity score models constructed separately for the new users and warfarin-experienced users.**  Analysis of impact of warfarin time in therapeutic range (TTR)  TTR was higher in the CPRD cohort than in ARISTOTLE (mean 0.73 vs. 0.62, median 0.76 vs 0.66).  Analysis by TTR subgroup suggested superiority non-inferiority of apixaban vs warfarin in those with TTR < 0.75 [Stroke/SE ~~0.88 (0.67 to 1.17)~~ **0.94 (0.75,1.19),** all-cause death ~~0.84 (0.73 to 0.98)~~ 0.99 **(0.87,1.12)**] (Figure 23). Apixaban was associated with increased hazards of both outcomes compared to warfarin in those with well-controlled warfarin treatment (TTR ≥ 0.75) [Stroke/SE ~~1.86 (1.38 to 2.52)~~ **1.49 (1.13,1.97)**], all-cause death ~~2.18 (1.84 to 2.60)~~ **1.75 (1.49,2.06)]** (Figure 3).  Safety results  Analysis by TTR ~~subgroup~~ suggested superiority of apixaban for major bleeding in those with TTR <0.75 [**0.70 (0.59,0.82)** ~~0.67 (0.55 to 0.81)~~] whereas apixaban users had a higher risk of major bleeding compared with those with optimal warfarin control (TTR ≥ 0.75) [**1.31 (1.08,1.59)** ~~1.45 (1.17 to 1.80)~~].  Figure 3 and Figure 4 values and captions updated  Discussion  ARISTOTLE presented outcomes by centre (for example hospital) TTR which did not show a signal of treatment efficacy of apixaban vs. warfarin differing by centre TTR quartile. We were able to exploit the use of ~~1:1 propensity score matching~~ **inverse probability of treatment weighting** to stratify our population based on TTR **whilst maintaining balance in baseline covariates targeting the treatment effect in the different warfarin TTR groups**. This showed that in patients well-controlled on warfarin (TTR ≥ 0.75) warfarin is superior to apixaban whereas in patients not well-controlled on warfarin (TTR < 0.75) apixaban is ~~superior~~ **non-inferior**. |
|  | The wording of the main conclusion in the abstract did not seem to match the results on first reading. Yes, the results showed non-inferiority, as did ARISTOTLE, but clearly not superiority, which ARISTOTLE did. To say the results are comparable therefore seems incongruous. To describe the treatment effect observed as "weaker" doesn't seem to fit. Looking at the effect estimates and CIs, I would describe the results as showing no treatment effect in the full population. The observation that this is a combination of a treatment effect benefit in the TTR<0.75 group and a treatment harm in the TTR>=0.75 group then makes sense. | Thank you for drawing attention to this discrepancy between the results and abstract conclusion. The conclusion in the abstract has been amended to reflect the results:  “Analysis of non-interventional data generated results ~~comparable to ARISTOTLE~~ **demonstrating non-inferiority of apixaban vs warfarin consistent with the pre-specified benchmarking criteria**. ~~The weaker treatment benefit~~ **Unlike in ARISTOTLE, superiority of apixaban vs warfarin was not seen** which may be linked to the **lower proportion of Asian patients and** higher proportion of patients with well-controlled warfarin compared to ARISTOTLE. The methodological template developed can be used to investigate treatment effects of oral anticoagulants in patient groups excluded from or under-represented in trials.” |
|  | Finally, and I don't know enough about the theory to be definitive on this, but I have read that propensity score matching can introduce bias rather than remove it. What assurances can the authors give that the way they have implemented PSM is good? Does it come down to the choice of caliper width? | I agree the dropping of patients involved in PSM has the potential for bias; coarsened exact matching (CEM) and IPTW were explored as alternative methods. CEM was found to be not feasible to replicate ARISTOTLE due to insufficient sample sizes in the subgroups to enable matching on all key variables. Whilst IPTW has the advantage of not dropping patients, the methods incorporating prevalent new user designs that were trialled had been proposed using propensity score matching. For the objective of replicating an RCT propensity score matching performs well at emulating the randomisation in an RCT. The impact of dropping patients is limited in this study given only a low proportion of patients were not matched successfully (274/9120 = 3.0% ARISTOTLE-matched apixaban users with no match).  Text added to Results of Propensity score matching: “**From 9120 apixaban users only 274 (3.0%) were dropped due to unsuccessful matching**.”  Text on this limitation (use of PSM) has been added to the manuscript:  “**The use of propensity score matching has the potential to introduce bias by dropping patients from the cohort [19], however propensity score matching is well suited to the process of trial emulation including prevalent users and a low number of apixaban users were dropped due to unsuccessful matching.”**  Future work could explore the use of IPTW when including prevalent users, for example by applying IPTW to the pool of prevalent users sampled on prior treatment history and calculating weights within the different strata.  The quality of PSM can be assured from the balance of the baseline variables in the matched cohort shown in Table 3 Baseline Characteristics in which a low standardised difference can be seen for all variables. Typically, a threshold of <0.1 in standardised difference is considered as represented adequate balance on baseline variables with perfect balance represented by standardised difference of 0 on all variables.  As recommended in the literature (eg see brief overview from Bergstra et al doi: 10.1136/rmdopen-2019-000953) the process of PSM was iterated by trialling different propensity score models, including inclusion of interaction and higher order terms, to optimise baseline balance. Balance on the interaction between the different stroke risk factors was a key focus in this study given the literature in this area (ie it is not only the total numerical stroke risk score which should be balanced but rather the particular combinations of stroke risk factors which can have different stroke risks despite having the same CHADS2 score). |
| **Reviewer #3** | | |
| 1 | Introduction: It is recommended to provide a more detailed explanation of how the results would remain consistent or similar to RCT results, even when the population that was excluded from or underrepresented in trials is included. In this regard, the 'validation of results against ARISTOTLE' section in the Methods section may need further elaboration for the benefit of readers. | Thank you for highlighting the ambiguity in the text. The validation criteria of results against ARISTOTLE have been moved from the appendix into the main text and the qualifier “in the trial-analogous CPRD cohort” have been added to make it clear that this validation step applies only to the primary effectiveness outcome in the trial-matched group in which we would expect to observe similar results to the trial on account of the similarity of the patients included (trial eligibility criteria applied and matched to the trial on baseline characteristics).  The results would not necessarily be expected to remain consistent or similar to the RCT results when looking at patient groups excluded from or under-represented from the trial as we may expect their relative risks to differ (eg patient groups with higher bleeding risk may see more benefit of apixaban over warfarin when compared with the RCT). A sentence has been added to the 'validation of results against ARISTOTLE' section to clarify this point:  **The validation step applied only to the primary effectiveness outcome in the trial-analogous CPRD cohort; results in other groups such as patients underrepresented or excluded from the trial would not necessarily be expected to remain consistent to the RCT results given the relative risks may differ in these groups. Comparability of other outcomes was to be assessed descriptively with no formal criteria used.** |
| 2 | Methods: For the definition of a new user of warfarin or a warfarin-naïve apixaban user, is there a required washout period to ensure their non-use of warfarin for a certain duration? | New users of warfarin or warfarin-naïve apixaban users were required to have no evidence of prior exposure to VKA in the 5 years prior to the index date. This detail has been added to the exposures section. |
| 3 | Methods: Why was a caliper of 0.2 chosen for use? | A caliper width of 0.2 times the pooled estimate of the common standard deviation of the logit of the propensity score was used as recommended by Austin (2009). Text and reference has been added to the manuscript to clarify this point:  **Step 3: matching of apixaban trial-analogous patients to warfarin trial-eligible patients in CPRD**  “**The trial-analogous CPRD Aurum apixaban patients were matched to warfarin CPRD Aurum patients using greedy nearest-neighbour matching on the logit of the propensity score (PS); a caliper of 0.2 times the standard deviation of the logit of the propensity score was used for matching as recommended by Austin [22].**“ |
| 4 | Results: 354 apixaban patients who had no matches were mentioned, along with 8846 patients who remained after matching. The numbers do not align (9120 - 354 = 8766). | We apologise there was a mistake in the numbers in the text – the correct number of apixaban patients with no matches is 274 (per flow chart in figure 2 we have 10 VKA-naïve with no match + 264 VKA-experienced with no match giving a total of 274). The numbers then align (9210 - 274 = 8846). I have updated the numbers in the text to the correct number (in Results Participants, 2^nd^ paragraph). |
| 5 | Discussion: In an RCT, participants are expected to maintain good adherence to their assigned treatment. However, in this study, I couldn't find a clear operational definition or justification for 'persistent patients' (e.g., checks at every 6-month time point would suffice?), 'discontinuation' (e.g., a definition using the number of days between prescriptions?), and 'switching' (e.g., when a prescription for a switching drug occurred during or certain days after the index treatment's prescription, how should this be considered?). It would be beneficial to provide further elaboration regarding these in both the Methods and Discussion sections. | Thank you for drawing attention to this, text has been added to define all these terms in the sensitivity analyses section in the methods section:  **Treatment persistence was defined by looking at longitudinal prescription data for OACs; OAC treatment windows were derived in which gaps >= 6 months between prescription dates were considered as distinct treatment windows. The end of each OAC treatment window was derived as the date of the last prescription of index OAC + the number of days supply given in the last prescription + a grace period of 30 days. In cases of overlapping OAC treatment windows the date of the first prescription of the subsequent OAC treatment window was used to define the end of the prior OAC window. A prescription for a different OAC from the index OAC treatment was considered as a treatment switch. An ending of index OAC treatment with no subsequent prescription for any other OAC recorded was considered as treatment stop.**  **Gaps of >= 6 months with no subsequent OAC prescriptions recorded were categorised as having stopped OAC treatment.**  In addition detail on derivation has been added to the footnotes of the tables in the appendix relating to treatment persistence. |
| 6 | Discussion: Is emulating an RCT in 2014 still relevant for today's real-world patient populations, given the changing preferences of patients and physicians, as well as evolving clinical practices, guidelines, and recommendations? | We agree the time period is a limitation and in fact posed a challenge in that there was a rapid uptake of apixaban as first line OAC, this has been added to the limitations section. In the context of increasing availability and use of real-world data the broader questions on ability to emulate an RCT in this therapeutic area, comparability of results between RCT and UK EHRs, and the methodological challenges in doing so are considered of value to inform future comparative effectiveness studies. The remaining differences observed between our cohort and the RCT population are of consideration for future RCT assessments when considering the relevance of RCT results to the UK or European population in the context of expected differences (in this case difference of TTR and ethnicity observed in the RCT versus the population in UK).  Emulating an RCT in 2014, and considering that our study included data from patients starting treatment up to July 2019, is more relevant for today’s real world patient populations to assess if trial results are generalisable to current UK populations instead of relying on out-dated evidence. Our study does not only aim at emulating an RCT, but also looks into other factors in real-world setting such as TTR and therefore, our study has both the merits of good validity and high generalisability to real-world clinical setting.  With specific reference to the clinical area of our study, we note that whilst OACs were rapidly taken up as a treatment for AF, warfarin is still in use, and therefore comparative evidence of their effects will be of interest and value to clinicians and patients. |
| 7 | Discussion: Could we explore future analyses with an extended follow-up period stemming from this study, particularly for the purpose of projecting long-term outcomes from an RCT? Are there any other valuable directions or extensions for this study that we should consider? | We agree that an extended follow-up time could be of interest with the limitation of an increased impact of attrition bias in the warfarin arm as the duration of follow-up is increased. Methods such as inverse probability of censor weighting should therefore be applied to extended analyses to account for the attrition bias. We recommend to look at longer duration of follow-up and have added a sentence to the discussion..  Discussion, paragraph 4 sentence added:  “**We recommend future analyses with an extended follow-up period from this study to compare the long term outcomes seen in the non-interventional cohort with projected long-term outcomes from the RCT**.” |
| Additional updates related to trial emulation terminology | | |
| 1 | During preparation of these responses, our team has been working in parallel on trying to improve understanding of the strengths, weaknesses and reporting requirements of studies that specifically aim to emulate an **existing** RCT in observational data and then benchmark observational results against RCT results (as we have done here). In particular, this is to distinguish this type of approach from that in which design is based solely upon a **hypothetical** target trial, by using the term “reference trial emulation” when design is based upon and benchmarked against an existing RCT.  A commentary pre-print on this topic can be found here [ref to pre-print], as can another pre-print analysis paper using this terminology (https://www.medrxiv.org/content/10.1101/2024.01.17.24301397v1.full.pdf) – both of these articles are currently under review at other journals. A third article using the reference trial emulation terminology has been accepted for publication in another journal.  In order to be consistent with this proposed use of terminology and improve the understanding of the approach used, we have made updates to the manuscript in relation to use of the term reference trial emulation as detailed here. | **Updated title:**  “Comparison of oral anticoagulants for stroke prevention in atrial fibrillation: a cohort study in the UK Clinical Practice Research Datalink with emulation of a reference trial (ARISTOTLE)”  This update incorporates suggested editorial changes to the original title as well as including that a reference trial emulation design has been applied and what the reference trial was called.  **Abstract**  “warfarin from 1 Jan 2013 to 31 Jul 2019 **were selected**” (stylistic change unrelated to trial emulation approach that we’d like to make)  “results **benchmarked against** the trial results”  **Introduction**  Line 148  “However, **reference trial emulation involves use of an existing named RCT to (1) inform observational study design and (2) benchmark results against, providing confidence in validity of the selected observational methods and data** [10-13]. The non-interventional analysis methods can then be applied, under a set of assumptions, to reliably estimate effects in groups of AF patients with AF who would have been excluded from (or underrepresented in) **the reference trial** [13].“  **Study design**  “A propensity score matched cohort study **emulation of a reference trial (ARISTOTLE).** “  Methods Line 363  “**Benchmarking results against ARISTOTLE**  The study hypothesis was that results in the CPRD ARISTOTLE-analogous cohort would be comparable to the ARISTOTLE results, as defined by the pre-specified benchmarking criteria.“  Methods Line 369  “The **benchmarking** criteria for considering…“  **Conclusions**  Line 647  “**Applying a reference trial emulation approach allowed us to** ~~Emulation of~~ **emulate a** landmark randomized trial ~~comparing between~~ of apixaban ~~and~~ **versus** warfarin ~~using~~ in non-interventional data, **with** ~~generated~~ results ~~consistent with~~ meeting pre-specified ~~validation~~ **benchmarking criteria based on** **the reference** trial **results**.“ |
