# Supplementary material for: Comparison of oral anticoagulants for stroke prevention in atrial fibrillation using the UK clinical practice research Datalink Aurum: A reference trial (ARISTOTLE) emulation study
Source: PLoS Med. 2024 Aug 29;21(8):e1004377. doi: 10.1371/journal.pmed.1004377 (PMC11361421; doi:10.1371/journal.pmed.1004377)
Supplement: Attachment — Submitted filename: Responses_to_review_15Mar2024.docx [file pmed.1004377.s005.docx]

PLOS Medicine

March 15^th^ 2024

To the Editor,

**RE: Manuscript PMEDICINE-D-23-02702R1 Decision - [EMID:21c59127b039996a]**

Please find as an attachment to this letter an annotated response to reviewers’ comments to the revised submission. We also provide the amended manuscript (one version with changes marked, one clean copy).

Your Sincerely,

Emma Powell
Faculty of Epidemiology and Population Health
London School of Hygiene and Tropical Medicine
Keppel Street, London WC1E 7HT, UK
[maud.teoh@lshtm.ac.uk](mailto:maud.teoh@lshtm.ac.uk)

**Response to review**

We very much appreciate the feedback provided. The original comments are included in the “feedback” column, while full details of our responses and any changes made (or reasons for not making changes) provided in the “Response and details of any changes made” column.

| **#** | **Feedback** | **Response and details of any changes made** |
| --- | --- | --- |
|  |  | Please note update to the affilations of one of the co-authors.  This also requires to add a disclaimer which I have placed after affiliation, however I was not sure if this was the correct location?  ‘The views expressed in this paper are those of the author and not do not necessarily reflect those of the SFDA or its stakeholders. Guaranteeing the accuracy and the validity of the data is a sole responsibility of the research team.’ |
| Requests from Editors GENERAL | | |
| 1 | TITLE  Suggest emphasizing more clearly the fact that this is target trial emulation study. Perhaps,  “Comparison of oral anticoagulants for stroke prevention in atrial fibrillation using the UK Clinical Practice Research Datalink Aurum: A reference trial (ARISTOTLE) emulation study”. | Thank you for the suggestion, title updated. |
| 2 | ABSTRACT  Abstract Background:  We appreciate the inclusion of additional detail which is very helpful to ascertain the aim of your study. For the purpose of brevity suggest removing lines 25-27 from the abstract and restricting to the introduction of main manuscript. Suggesting including a final sentence in the 1st paragraph of ‘Background’ to read as follows:  ‘Reference trial emulation allows evidence to be generated around treatment effects in groups excluded or underrepresented in the original trials.’  Abstract Methods & Findings:  Line 37 – suggest revising ‘warfarin CPRD users’ to ‘warfarin users in the CPRD Aurum’.  Abstract Conclusions:  Do these data tell us anything about the usefulness of this methodological approach to investigate treatment effects for other conditions? | Thank you for the comments.  Abstract Background: Updated to remove lines 25-27 as suggested.  Final sentence in 1^st^ paragraph added: ‘Successful reference trial emulation can support the generation of evidence around treatment effects in groups excluded or underrepresented in the original trials’  Abstract Methods & Findings: Updated as suggested.  Abstract Conclusions: Text added  ‘The methodological template developed can be used to investigate treatment effects of oral anticoagulants in patient groups excluded from or under-represented in trials and also provides a framework which can be adapted to investigate treatment effects for other conditions.’ |
| 3 | AUTHOR SUMMARY  Line 65 – suggest, ‘This study used routinely collected health data from the UK to…’  Lines 69-70 – suggest, ‘…help to understand how transferrable RCT results are to ‘real-world’ practices and whether this methodological approach can help to improve treatment options and outcomes for patient groups currently underrepresented in clinical trials.’ Or similar.  Line 75 – suggest, ‘UK primary care data’.  Line 81 – sentence beginning ‘This may be explained…’ suggest removing and (after paraphrasing) suggest placing as part of the final bullet point of the ‘What do these findings mean?’ sub-section as a limitation.  Lines 88 and 90 – suggest swapping these 2 bullet points around such that the ‘benefits’ of the methodological approach are listed sequentially.  Line 93 – suggest removing the word ‘these’ as this methodology could be applied to various treatment strategies for a number of different conditions – this would be worth emphasizing as it is a major selling point of your study, at least in my opinion! Might also be worth giving an example and making a clear distinction between those that are excluded from clinical trials (those with multimorbidity, for example) and those who are underrepresented (certain ethnic minority groups, for example).  Line 96 – please revise the final point in line with previous comments (see line 81). | Line 65 updated as suggested.  Line 69-70 updated as suggested.  Line 75 updated as suggested.  Line 81 – sentenced removed and final bullet point of ‘What do these findings mean?’ sub-section amended:  ‘Study limitations include the possibility of residual confounding ~~and~~, a risk patients doing well on warfarin were over-represented in our cohort, and a lower proportion of Asian participants in our cohort compared with ARISTOTLE.’  Lines 88 and 90 bullet points swapped.  Line 93 ‘these’ removed.  Examples added:  ‘We can use the data and methods to look at how well treatments work in patients that would not have been included in RCTs such as those with multimorbidity or patient groups under-represented in RCTs such as ethnic minority groups and older patients.’  Last bullet point: added text ‘, and a lower proportion of Asian participants in our cohort compared with ARISTOTLE ‘ |
| 4 | INTRODUCTION  As noted above, the methodological approach is a really big selling point which deserves emphasis but not only for anticoag. in AF, also for a variety of treatments and differing conditions.  As above, might be worth differentiating & defining the excluded and underrepresented groups so that readers can make tangible associations, which should increase the impact of your take-home message.  Line 140 – would ‘influenced by’ be more accurate than ‘dependent upon’? | Added text to the introduction:  ‘There is increasing interest in trial emulation using observation data, and in the application of recent developments in pharmacoepidemiology methods involving the inclusion of prevalent users. This study used a framework which involved coarsened exact matching to select patients matching the trial population on aggregate and sampling of prevalent users in a way that avoids selection bias and emulates the process of screening into an RCT, offering a way of constructing a cohort of patients similar to the target trial population which included both new and prevalent users. This methodological approach could be adapted to a variety of treatments and different therapeutic areas.’  Added text giving examples for both:  ‘The non-interventional analysis methods can then be applied, under a set of assumptions, to reliably estimate effects in groups of patients with AF who would have been excluded from (or underrepresented in) the reference trial [14], such as patients aged > 80 that were under-represented in ARISTOTLE compared with patients with AF in UK clinical practice and patients with increased bleeding risk that were excluded by the trial eligibility criteria.’  Line 140 updated as suggest to ‘influenced by’. |
| 5 | TABLES and FIGURES  Table 3 – for the purpose of formatting requirements, please include a leading zero for all numerical values in the standardized difference column, for example, row 3 should read ‘0.008’. If not for the purpose of transparent reporting, please clearly explain the reasons why not. | Leading zero added to all numbers in the standardised difference column. |
| 6 | DISCUSSION  Some parts of your discussion read very well and others are less nuanced and focused. Suggest revising for improved nuance and clarity. The strengths and limitations of your findings in context of your chosen methodological approach, which we think has clear strengths and transferability as well as some limitations, could be more clearly presented in parts.  Please remove all sub-headings such that the discussion reads as continuous prose.  Lines 528-530 – when referring to ‘prevalent users’ do you mean users of warfarin, apixaban or both. Please amend for clarity.  Line 568 onwards – you discuss here the implications of ethnicity on your results compared to ARISTOTLE. For further clarity, could you elaborate on the reasons why such a difference was observed. Was this a consequence of the database or an effect of PSM? Considering that this (methodological) approach could help in ascertaining treatment effects in those who are underrepresented in trials (such as ethnic minority groups), what implications might this finding have on the usefulness of either the dataset or the methodological approach more broadly? | Discussion section revised for improved nuance and clarity (including additional detail in first paragraph, re-ordering of text, removal of repetition, additional references.  Subheadings removed from discussion and text added at the start of paragraphs instead to aid identification of strength, limitations and conclusion.  Text added to clarify meaning of prevalent users:  ‘~~We found using~~ A key strength of our study was the use of a framework which sample~~s~~d prevalent users (the continuing users of warfarin in this study) in a way that avoid~~s~~ed selection bias o~~ffers a way of~~ facilitating the constructi~~ing~~ion of a cohort of patients similar to the target trial population, which included both new users of apixaban and warfarin (VKA-naïve) and patients with prior VKA exposure (VKA-experienced) that were randomised to stay on warfarin or switch to apixaban.’  Text added:  ‘This limitation on ethnicity arose from the data source used and time period studied (patients with AF in CPRD Aurum 2013-2019) which had a low proportion of Asian patients, likely due to AF being associated with older age combined with a lower prevalence of AF in Asian patients compared with White patients [38]. Whilst CPRD Aurum is largely representative of the UK population in relation to ethnicity [39], diversity is still limited for older individuals. Despite this, CPRD Aurum has shown to be a useful resource for investigating treatment effects in different ethnic groups for indications such as hypertension which is more prevalent and occurs at a younger age in ethnic minority groups, with similar trial replication methods used to compare antihypertensive treatment effects in underrepresented ethnic groups [40]. |
| 7 | SUPPORTING INFORMATION  Please ensure that abbreviations are defined throughout the supporting information, including tables and figures as relevant.  Thank you for including the published protocol. Apologies for the confusion at the time of our previous request we were asking for the original protocol document (i.e., as used for your study approval process or data access process) not the BMJ published protocol article, this does not need to be included, the reference will suffice. Please amend. | Definitions added throughout supporting information (appendix S3) and references added within S3.  Appendix S2 updated to be ISAC protocol submitted to the MHRA for access to CPRD data. References to published protocol and appendix updated in the manuscript:  The use of CPRD and ARISTOTLE are described in a previous publication [14] and use of CPRD for this project was approved by the MHRA Independent Scientific Advisory Committee [ISAC protocol in S2]. All data used in this study were anonymised. |
| 8 | SOCIAL MEDIA  To help us extend the reach of your research, please detail any X (formerly Twitter) handles you wish to be included when we tweet this paper (including your own, your coauthors’, your institution, funder, or lab) in the manuscript submission form when you re-submit the manuscript. | Please include the following X handles:  @LSHTM, @ehr_lshtm, @The_MRC,  @JohnTStats, @Paris_Baptiste1, @TurkiMo94 |
|  | Comments from Reviewers: |  |
|  | Reviewer #2: Alex McConnachie, Statistical Review  I thank the authors for their consideration of my original comments. I am happy with their responses, and have no further comments to make. | Thank you for your review. |
