# Supplementary material for: Comparison of oral anticoagulants for stroke prevention in atrial fibrillation using the UK clinical practice research Datalink Aurum: A reference trial (ARISTOTLE) emulation study
Source: PLoS Med. 2024 Aug 29;21(8):e1004377. doi: 10.1371/journal.pmed.1004377 (PMC11361421; doi:10.1371/journal.pmed.1004377)
Supplement: Attachment — Submitted filename: Responses_to_review_30May2024.docx [file pmed.1004377.s006.docx]

PLOS Medicine

May 30^th^ 2024

To the Editor,

**RE: Manuscript PMEDICINE-D-23-02702R1 Decision - [EMID:21c59127b039996a]**

Please find as an attachment to this letter an annotated response to reviewers’ comments to the revised submission. We also provide the amended manuscript (one version with changes marked, one clean copy).

Your Sincerely,

Emma Powell
Faculty of Epidemiology and Population Health
London School of Hygiene and Tropical Medicine
Keppel Street, London WC1E 7HT, UK
[maud.teoh@lshtm.ac.uk](mailto:maud.teoh@lshtm.ac.uk)

**Response to review**

| **#** | **Feedback** | **Response and details of any changes made** |
| --- | --- | --- |
| Editors | | |
| 1 | N/A | The abstract has been modified to comply with the word limit (15 words removed):  Abstract  ‘’ Stroke prevention ~~treatment~~ guidance for patients with atrial fibrillation (AF) uses evidence generated from randomised controlled trials (RCTs).  … however there remains uncertainty on the validity of ~~the~~ methods and suitability of the data.  Successful reference trial emulation can support the generation of evidence around treatment effects in groups excluded or underrepresented in ~~the original~~ trials. … Patients with AF in the ~~a~~UK ~~primary care database~~ Clinical Practice Research Datalink (CPRD Aurum) prescribed apixaban or warfarin from 1 Jan 2013 to 31 Jul 2019 were selected. … The ~~analysis sample~~dataset comprised 8734 apixaban users and propensity-score matched 8734 warfarin users ~~in CPRD~~. … Analysis of non-interventional data generated results demonstrating non-inferiority of apixaban vs warfarin consistent with ~~the~~ pre-specified benchmarking criteria. Unlike in ARISTOTLE superiority of apixaban vs warfarin was not seen, possibly due ~~which may be linked~~ to the lower proportion of Asian patients and higher proportion of patients with well-controlled warfarin compared to ARISTOTLE. This~~e~~ methodological template ~~developed~~ can be used to investigate treatment effects of oral anticoagulants in patient groups excluded from or under-represented in trials and ~~also~~ provides a framework which can be adapted to investigate treatment effects for other conditions.” |
|  |  |  |
| Reviewer #2: Alex McConnachie, Statistical Review | | |
| 1 | Selection bias and immortal time bias in the analysis by TTR (regarding suggestion to perform an analysis by prior TTR) | We performed an analysis by prior TTR however we found this analysis to be limited by the small number of patients with prior TTR available (subset of the patients with prior VKA exposure): Outcome TTR prior to index date category \| Apixaban Event Rate %/yr \| Warfarin Event Rate %/yr \| Hazard Ratio (95% CI); TTR < 0.75 \| (N=1919) \| (N=1919) \|; TTR ≥ 0.75 \| (N=1788) \| (N=1788) \|; Stroke or systemic embolism \| \| \|; TTR < 0.75 \| 1.71 \| 1.52 \| 1.12 (0.79, 1.61); TTR ≥ 0.75 \| 1.07 \| 1.37 \| 0.81 (0.49, 1.35); Ischemic stroke \| \| \|; TTR < 0.75 \| 1.31 \| 0.83 \| 1.59 (1.01, 2.48); TTR ≥ 0.75 \| 0.81 \| 0.93 \| 0.90 (0.50, 1.64); Hemorrhagic stroke \| \| \|; TTR < 0.75 \| 0.21 \| 0.22 \| 0.91 (0.34, 2.41); TTR ≥ 0.75 \| 0.29 \| 0.33 \| 0.96 (0.39, 2.36); All-cause death \| \| \|; TTR < 0.75 \| 7.72 \| 7.39 \| 1.04 (0.88, 1.22); TTR ≥ 0.75 \| 4.55 \| 4.40 \| 1.03 (0.77, 1.36); Major bleeding \| \| \|; TTR < 0.75 \| 2.87 \| 3.48 \| 0.82 (0.62, 1.07); TTR ≥ 0.75 \| 2.73 \| 2.93 \| 0.94 (0.65, 1.35) ARISTOTLE performed an analysis of on-treatment TTR relying on grouping by site to balance baseline variables by treatment arm. We therefore think an analysis by on-treatment TTR is of more interest. |
| 2 | Selection bias and immortal time bias in the analysis by TTR (regarding update to methods) | To address the selection bias and immortal time bias that is introduced by restricting the analysis to those patients with TTR measurement available the analysis was updated to include all warfarin users. For patients on warfarin with TTR missing we predicted their TTR category based on their baseline variables. The model was constructed used those that were not missing TTR with performance of the model assessed as the proportion of concordant pairs (predicted TTR category vs acutal TTR category). INR values were restricted to the first year after index date to attempt to minimise selection bias. Two different models were trialled for prediction of TTR: a model for continuous TTR and a logistic regression model modelling TTR categorised as high (TTR >= 0.75) or low (TTR < 0.75). The logistic regression model successfully predicted the largest proportion of concordant pairs (observed TTR category vs predicted category) and was therefore selected to be used to predict TTR for patients missing TTR. Baseline variables included in the prediction of TTR model (literature on prediction of TTR was used to guide model selection along with iterative improvement keeping only significant predictors and best model fit): age, sex, BMI, smoking status, diabetes, congestive heart failure, statins, ACEi or ARB, beta-blockers, digoxin, amiodarone, NSAIDs, PPI, prior VKA exposure [naïve, <6 months prior exposure, >= 6 months prior exposure], alcohol consumption, IMD2015_5, renal function, COPD. Once predicted TTR category had been derived for patients missing TTR the analysis by TTR was re-run using the same method as used in the original analysis (ie applying IPTW to the apixaban users to target the treatment effect in the warfarin TTR group). Summary of updated results (full results tables in updated appendix): Outcome TTR category \| Apixaban Event Rate %/yr \| Warfarin Event Rate %/yr \| Hazard Ratio (95% CI); Stroke or systemic embolism \| \| \|; TTR < 0.75 \| 1.36 \| 1.47 \| 0.91 (0.73, 1.14); TTR ≥ 0.75 \| 1.15 \| 1.11 \| 1.05 (0.82, 1.34); Ischemic stroke \| \| \|; TTR < 0.75 \| 0.95 \| 0.94 \| 1.00 (0.76, 1.32); TTR ≥ 0.75 \| 0.85 \| 0.67 \| 1.24 (0.92, 1.68); Hemorrhagic stroke \| \| \|; TTR < 0.75 \| 0.23 \| 0.36 \| 0.63 (0.38, 1.04); TTR ≥ 0.75 \| 0.20 \| 0.31 \| 0.72 (0.43, 1.21); All-cause death \| \| \|; TTR < 0.75 \| 5.05 \| 5.27 \| 0.94 (0.84, 1.06); TTR ≥ 0.75 \| 3.75 \| 3.13 \| 1.20 (1.04, 1.37); Major bleeding \| \| \|; TTR < 0.75 \| 2.66 \| 3.54 \| 0.74 (0.63, 0.86); TTR ≥ 0.75 \| 2.22 \| 2.07 \| 1.08 (0.90, 1.30) Manuscript updated throughout with updated results for the analysis by TTR: Abstract ‘Analysis by TTR suggested similar effectiveness of apixaban compared with poorly controlled warfarin (TTR < 0.75) for [Stroke/SE [0.91 (0.73, 1.14)], all-cause death 0.94 (0.84, 1.06)], and superiority for major bleeding [0.74 (0.63, 0.86)]. However, when compared with well controlled warfarin (TTR ≥ 0.75) apixaban was associated with an increased hazard for all-cause death [1.20 (1.04, 1.37)] and there was no significant benefit for major bleeding [1.08 (0.90, 1.30)]…’ Author summary ‘• We also found the benefit of apixaban vs warfarin differed for some outcomes depending on the quality of warfarin therapy with apixaban (i) superior only to poorly controlled warfarin therapy for major bleeding [TTR < 0.75: Stroke/SE 0.74 (0.63, 0.86), TTR ≥ 0.75: 1.08 (0.90, 1.30)] (ii) associated with an increased risk of death compared only to well-controlled warfarin therapy [TTR ≥ 0.75: 1.20 (1.04, 1.37), TTR < 0.75: 0.94 (0.84, 1.06)].’ Supplementary analyses ‘An analysis by INR TTR was performed instead to assess the impact of warfarin control on results with all outcomes analysed by TTR (TTR < 0.75 and TTR ≥ 0.75). Individual predicted TTR based on baseline variables was used for patients missing TTR. In order to perform the TTR analysis whilst maintaining balance in the baseline covariates, inverse probability treatment weighting (IPTW) was used to rebalance the baseline characteristics, applying stabilised weights to the ARISTOTLE-analogous apixaban users. A similar approach to the main analysis was used with propensity score models constructed separately for the new users and warfarin-experienced users.’ Missing data ‘A total of 1176 (13.3%) warfarin users in the CPRD cohort did not have INR measurements in the data during their treatment period with predicted TTR used for these patients in the analysis by TTR (see Appendix for details).’ Main results Figure 3 updated Analysis of impact of warfarin time in therapeutic range (TTR): ‘Analysis by TTR suggested non-inferiority of apixaban vs warfarin in those with TTR < 0.75 [Stroke/SE 0.91 (0.73,1.14), all-cause death 0.94 (0.84, 1.06)] (Figure 3). Apixaban was associated with similar hazards for stroke by category of TTR and increased hazards of death compared to warfarin in those with well-controlled warfarin treatment (TTR ≥ 0.75) [Stroke/SE 1.05 (0.82, 1.34), all-cause death 1.20 (1.04, 1.37)] (Figure 3).’ Safety results Figure 4 updated ‘Analysis by TTR suggested superiority of apixaban for major bleeding in those with TTR <0.75 [0.74 (0.63, 0.86)] whereas apixaban users had a similar risk of major bleeding compared with those with optimal warfarin control (TTR ≥ 0.75) [1.08 (0.90,1.30)].’ Discussion Paragraph 1 ‘We found higher TTR in the patients using warfarin in our cohort compared with the warfarin arm of ARISTOTLE (median 0.76 vs 0.66). While our analysis by TTR showed non-inferiority of apixaban vs warfarin for our stroke or systemic embolism outcome, we observed an increased risk of death on apixaban compared with that in patients well-controlled on warfarin (TTR ≥ 0.75) but not when compared with those on poorly controlled warfarin. For major bleeding, while apixaban was superior when compared to those on poorly controlled warfarin, there was no difference when compared to those on well controlled warfarin.’ Paragraph 5 ‘An additional strength of our study was the ability to explore the quality of warfarin treatment in our cohort and the impact of INR control on the treatment effect estimates. Our finding that the benefits of apixaban vs warfarin for some outcomes depended on the quality of INR control in the warfarin arm answers questions raised in the NICE premeeting briefing which looked at apixaban in the NVAF population and noted the TTR seen in ARISTOTLE “may be lower than what is typical in UK clinical practice” and “apixaban compared with well-controlled warfarin (TTR 75% or more) may not be superior in the long term” [8]. ARISTOTLE presented outcomes by centre (for example hospital) TTR quartile and did not show a signal of treatment efficacy differing by centre TTR quartile. We were able to use inverse probability of treatment weighting to estimate the treatment effect in the different warfarin TTR groups and used predicted TTR for warfarin users missing TTR to attempt to limite the risk of selection bias.’ Last paragraph ‘The weaker overall treatment benefit observed in our cohort appears to be due to a higher proportion of patients with well-controlled warfarin in the UK clinical context, compared with the trial. Our exploratory analysis by TTR showed similar results for stroke and a greater benefit for apixaban for major bleeding compared with TTR<0.75; conversely a slightly higher risk of death was observed on apixaban compared with well-controlled warfarin.’ |
| 3 | Selection bias and immortal time bias in the analysis by TTR (regarding description of the potential bias/limitations) | The updates to the methods should have removed immortal time bias (given that warfarin users no longer need to survive to be included in either TTR group), and limited the impact of selection bias that was caused in the original analysis by excluding the patients with missing TTR. We have added text throughout the manuscript to emphasise the possibility of selection bias and misclassification in this analysis:  Abstract, final sentence of methods and findings text added:  ‘The main limitation of the study’s methodology are the risk of residual confounding, channelling bias and attrition bias in the warfarin arm, and selection bias and misclassification in the analysis by TTR.’  Author summary, final bullet point text aded:  ‘• Study limitations include the possibility of residual confounding, a risk patients doing well on warfarin were over-represented in our cohort, and a lower proportion of Asian participants in our cohort compared with ARISTOTLE, and the likelihood of residual selection bias/misclassification in the TTR analysis.’  Discussion – 11^th^ paragraph text added:  ‘Adherence to treatment was difficult to assess … The analysis of TTR is limited by this being a post-baseline measure available for only one treatment arm leading to a risk of selection bias in this analysis – patients with TTR available in the study may be more healthy than those without this measure given that patients have to survive and not be hospitalised to have INR measurements available in CPRD Aurum. The limitation of use of a post-baseline measurement available for one treatment arm was also evident in the RCTs of DOACs vs warfarin and is mitigated in our study through the use of IPTW and predicted TTR for patients that were missing TTR (using a model to predict TTR that used INR measurements restricted to the first year of follow-up). Given the risk of selection bias in the analysis by TTR and risk of miss-classification of TTR for those missing TTR, these results should be considered exploratory and interpreted with caution.’ |
